# Enantiomeric Variability of Distaminolyne A. Refinement of ECD and NMR Methods for Determining Optical Purity of 1-Amino-2-Alkanols

**DOI:** 10.3390/molecules24010090

**Published:** 2018-12-27

**Authors:** A. Norrie Pearce, Brent R. Copp, Tadeusz F. Molinski

**Affiliations:** 1 School of Chemical Sciences, University of Auckland, Private Bag 92019, Auckland 1142, New Zealand; n.pearce@auckland.ac.nz (A.N.P.); b.copp@auckland.ac.nz (B.R.C.); 2 Department of Chemistry, University of California, San Diego, 9500 Gilman Drive MC0358, La Jolla, CA 92093, USA; 3 Skaggs School of Pharmacy and Pharmaceutical Sciences, University of California, San Diego, 9500 Gilman Drive MC0358, La Jolla, CA 92093, USA

**Keywords:** circular dichroism, amino alcohols, absolute configuration, marine natural products

## Abstract

Sample configurations of distaminolyne A (**1a**); isolated from the ascidians *Pseudodistoma opacum* and *P. cereum*, and collected at different sites in New Zealand, were investigated by two methods: Exciton coupled electronic circular dichroism (EC ECD) of the corresponding *N*,*O*-dibenzoyl derivative **1b**; and chiral reagent derivatization of **1a** with (*S*)- and (*R*)-α-methoxyphenylacetic acid (MPA), followed by ^1^H-NMR analysis. Configuration and optical purity of **1a** (%ee) was found to vary depending on the geographic distribution of ascidian colonies. An improved method for preparing *N*,*O*-diarenoyl derivatives of **1a** was optimized. The EC ECD method was found to be complementary to the MPA-NMR method at different ranges of %ee.

## 1. Introduction

The natural products sphingolipids—characterized by homologs and highly-modified analogs of the canonical C_18_ long-chain base, d-*erythro*-sphingosine—are rare among plants and microbes, but prevalent in the diverse realm of marine invertebrates [1]. The most common variations are 2-amino-alkane-1,3-diols and their ∆^4^-unsaturated derivatives, corresponding to **1** and d-*erythro*-4,5-dihydrosphingosine (sphinganine). Other variations include long-chain aminoalkanols (AAs), including 1-amino-2-alkanols (1-AAs), 2-amino-3-alkanols (2-AAs), and compounds that may be related to the latter through divergent biosynthetic pathways (e.g., long-chain 2*H*-azirines [2]). Many AAs are biologically active; for example, the simple homolog spisulosine from the clam *Spisula polynyma* inhibits cell proliferation by disassembly of actin stress filaments [3].

Unlike sphingosine, AA configurations are heterogeneous: The relative configuration (RC) and absolute configuration (AC) of 2-AAs vary from species to species. Configuration assignment to AAs is deceptively simple, and confounded by weak specific rotations that have been misinterpreted in the past leading to erroneous assignments [4], or no assignment, which defer the problem to total synthesis [5,6]. A single chirogenic center in 1-AAs is responsible for [α]_D_ with magnitudes within a narrow range of ~±1–3; for example, in *S*-distaminolyne A (**1a**, [α]_D_ −1), a C_17_ AA from the New Zealand ascidian *Pseudodistoma opacum* [7]. The configuration of **1a** was assigned from interpretation of exciton coupling observed in the electronic circular dichroism ECD spectrum of the corresponding *N*,*O*-dibenzoyl derivative **1b** [7] according to an extension of the exciton coupling circular dichroism (EC ECD) dibenzoate method pioneered by Nakanishi and Harada [8]. Prior to this report, the EC ECD method was applied successfully to **2b**, the *N*,*O*-dibenzoyl derivative of an unnamed 1-AA natural product; **2a**, from a didemnid ascidian collected on the Great Barrier Reef [9]. 

The configuration of (*S*)-**1a** was recently challenged by Sun et al. and ‘re-assigned’ as *R* based on a total synthesis of (*S*)- and (*R*)-**1a,** and the comparison of specific rotations—albeit of low magnitudes—with the published value of the natural product [10]. Paradoxically, a subsequent synthesis of **1a** by Dumpala et al. using different methodology, upheld the original 2*S* stereoassignment [11]. Notably, the validity of the EC ECD method used for the original assignments of **1b** [7] or **2b** [9] was not disputed—in fact, not even addressed—in either report, leaving a dilemma: which assignment is correct?

Upon revisiting the original assignment, we found the assignment of AC of (*S*)-**1a** was upheld by careful re-examination of the ECD data, and new comparisons to model 1-AAs, (*S*)-**3a**, and (*R*)-**3b** prepared by rational asymmetric synthesis, and converted to dibenzoyl derivatives **4a**,**b** [12]: The ECD spectrum of (*S*)-**1b** was essentially the same as that of (*S*)-**4a**. An elementary lesson in chiroptical analysis, reprised here, is that reassignment of natural products based solely on comparisons of [α]_D_ of weakly rotatory compounds is tenuous, at best, and unsupportable in light of stronger counter-evidence; for example, assignments based on the non-empirical interpretation of EC ECD [8]. 

In this report, we investigate chiroptical properties of additional acylates of 1-AAs, the curious chain-length dependence of diacylation reactions under standard conditions, and improved methods for preparing **1b** and related acylates. We also found configurational variability of **1a** isolated from samples of *P. opacum* and *P. cereum* collected at different sites from the North Island of New Zealand, and outline complementary methods for measuring AC and enantiomeric excess (%ee) of **1a** by ECD of *bis*-arenoyl derivatives, and ^1^H-NMR after conversion with a chiral derivatizing agent (CDA).

## 2. Results

**Figure 1:**
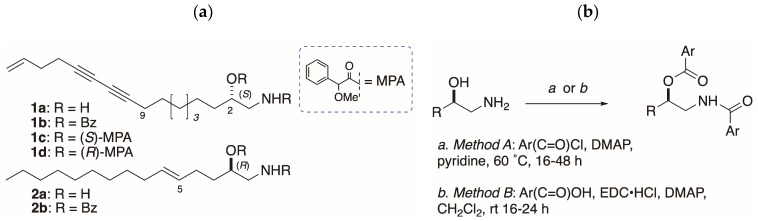
(**a**) Distaminolyne A (**1a**; [7]) and un-named 1-AA natural product (**2a**; [9]) and their corresponding acylates, **1b**–**d** and **2b**. (**b**) Two methods for acylation of 1-AAs.

Unlike acylation of 2-AAs, benzoylation of 1-AAs with BzCl-pyridine has delivered variable success, giving the desired dibenzoyl derivatives in low yields. For example, benzoylation of **1a** (Figure 1b; Method A: BzCl, 4 equiv, DMAP, pyridine, 50 °C, 64 h), gave **1b** in only 8%, but a larger amount (16%) of the mono-acylated *N*-benzamide, even with excess equivalencies of reagents [7]. Similar yields were obtained for model compounds (*S*)-**3a** and (*R*)-**3b**, giving (*S*)-**4a** (15%) and (*R*)-**4b** (25%; [12]). From earlier work, alternative conditions for benzoylation (*N*-benzoylimidazole, DBU, CH_3_CN, 60 °C [13]) applied to (2*S*,3*R*)-2-amin ododecan-3-ol, from *Clavelina oblonga* [14] or sphingolipids [15], were also less than satisfactory. Although it is certain that benzoylation of the amino group occurs as the first step, it was not clear why benzoylation of the secondary OH of the benzamide intermediate was so sluggish. One possibility is formation of micelles in the dipolar solvents pyridine, and CH_3_CN that retard the second benzoylation step (see below). 

### 2.1. Acylation of Model Compounds

**Figure 2:**
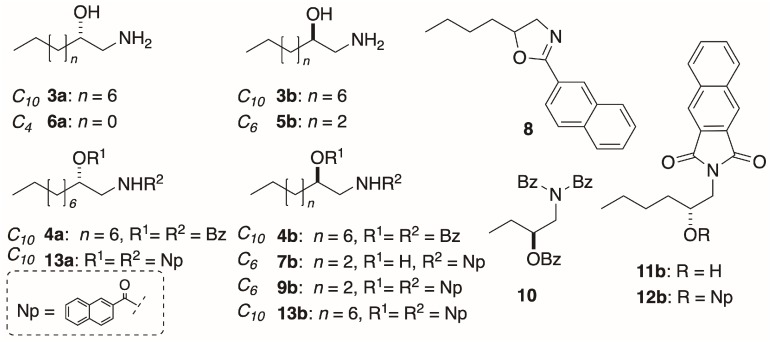
Model 1-amino-2-alkanols (1-AAs) **3**–**5** and their corresponding acylates.

In order to investigate the chain-length dependence and byproduct, we explored the acylation of homologous 1-AAs: The C_10_ compounds (*S*)-**3a** and (*R*)-**3b** (Figure 2), and the C_6_ and C_4_ homologs (*R*)-**5b** and (*S*)-**6a**, respectively. Naphthoylation of (*R*)-**4b** (Method A) gave mono *N*-naphthamide **7b** (Figure 2b) as the major product (18%), and smaller amounts of oxazoline **8** [0.4%, [α]_D_ +2.6 (*c* 0.5, CHCl_3_)], and the di-*N*,*O*-naphthoyl derivative (*R*)-**9b** (0.5%). Cyclodehydration of AA benzamides is known to occur with inversion of configuration at C-2 under certain conditions that activate the secondary OH group (e.g., Brønsted acid, SOCl_2_) for intramolecular nucleophilic *S*_N_2 displacement by the carboxamide oxygen [16,17]. Clearly, if a similar inversion was occurring at C-2 during formation of **1b** under elevated temperature and prolonged reaction times of Method A, it would undermine the validity of the ECCD assignment method for all 1-AAs. Likewise, if oxazolines were the intermediates on the pathway to **4a**,**b**, these model compounds would be invalid. In order to test this hypothesis, benzoylation of (*R*)-**3b** was carried out under the milder conditions (*Method B*; see below). Gratifyingly, the product (*R*)-**4b** showed an essentially identical specific rotation (${\left[\alpha \right]}_{D}^{23}$ −24.1 (*c* 1.48, MeOH)) to that of (*R*)-**4b** produced by Method A (${\left[\alpha \right]}_{D}^{23}$ −26.1 (*c* 1.78, MeOH); [12]) eliminating the possibility of inversion at C-2 under the latter conditions. 

Benzoylation of the shortest C4-chain 1-AA (*S*)-**6a** by Method A gave no dibenzoyl compound, but instead the *N*,*N*,*O*-tribenzoyl derivative (*S*)-**10b** as the only identifiable product (5%). Imide (*S*)-**10b** was deemed to be unsuitable for ECCD assignments due to the difficulty of establishing the directionality of the charge-transfer electronic transition dipole vectors of the three chromophores. 

Eventually, a more reliable method for *N*,*O*-dibenzoylation of **1a, 3a**, and **3b** was deployed (Method B: EDC∙HCl, benzoic acid, DMAP, 5 equiv. each, CH_2_Cl_2_, rt) that improved the yields of **1b**, **3a**, and **3b** (~40%–quant.) on a ~1–3 mg scale and suppressed acylation byproducts. For example, our earlier low yielding *N*,*O*-*bis*-(2-naphthoylation) of (*S*)-**3b** to (*R*)-**13b** (9%; [12]) was greatly improved by applying *Method B* with replacement of 2-naphthoic acid for benzoic acid: The 1-AA (*S*)-**3a** was smoothly converted to (*S*)-**13a** in 46% yield. 

A second approach for exciton coupling ECD analysis was evaluated based on Nakanishi’s reported microscale derivatization of amino-polyols for ‘picomole scale’ assignment of sphingosine bases through introduction of two different well-behaved chromophores in separate steps [18,19]. Primary amine (*R*)-**5b** was first converted to the naphthimide (*R*)-**11b** (pyridine, DMAP, naphthalene-2,3-dicarboxylic acid anhydride, 110 °C) in good yield (74%), followed by separate *O*-naphthoylation (2-naphthoic acid, EDC, DMAP) to give the derivative (*R*)-**12b** (72%). The CD spectra of the new chromophoric derivatives were measured and compared. 

Method B was also adapted to sub-µmole acylation of 1-AAs with modification of Nakanishi’s approach employing melting point capillaries as reaction vessels [18,19]. Treatment of **5b** (0.35 µmol; Method C) with a standard solution prepared from the reagents in DCE (2.8 equiv.; See Experimental) and heating (45 °C or 67 °C, 60 min; Method C) gave, after recovery and purification by preparative TLC, (*R*)-**12b** in yields of 31% and 40%, respectively (Calculated from measured absorbances at *λ*_max_ 231 nm (TFE)). 

### 2.2. Circular Dichroism of Acylated 1-AAs

**Figure 3:**
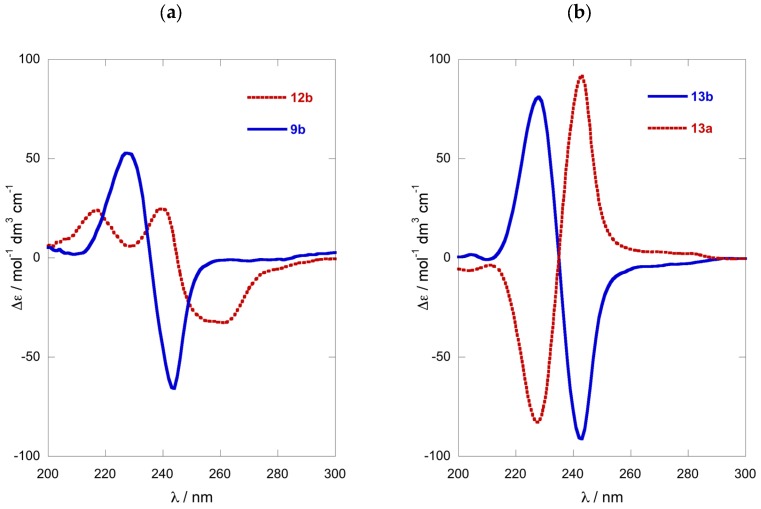
ECD spectra of: (**a**) (*R*)-**9b** (––) and (*R*)-**12b** (**---**) (TFE, 23 °C); and (**b**) (*S*)-**13a** (this work) and (*R*)-**13b** [12] (MeOH, 23 ˚C).

Comparison of the ECD spectra of the three shorter chain chromophoric derivatives of 1-AAs showed significant differences in magnitude and complexity. The CD spectrum of naphthoyl naphthimide (*R*)-**12b** (Figure 3a) displayed three major Cotton effects (CEs, *λ* 216 (∆ε +24.7), 240 (+25.8), and 259 (−31.8)) due to interaction of the 2-naphthoate charge-transfer (CT) band, likely with both the longitudinal CT band (^1^*B*_b_) and transverse bands (^l^*L*_a_, and ^1^*L*_b_) of the naphthimide chromophore in a complex manner [18]. In contrast, the ECD spectrum of dinaphthoyl derivative (*R*)-**9b** was relatively simple consisting of a split-Cotton effect (CE) at *λ* 228 (∆ε +53) and 244 (−66) of large magnitude (*A* = 119) from the two degenerate ^1^*B*_b_ CT bands. 

The ECD spectra of the (*R*)-*N*,*O*-bis-(2-naphthoyl) derivatives of C_6_ and C_12_ 1-AAs displayed ECD spectra of the same form (negative split CE), with only slight differences in magnitudes (Figure 3a,b). Compared to the longer C_10_ chain compound (*R*)-**13b** (*A* = 172, *A* is defined as the difference of ∆*ε* measured from trough to crest in the split-CE [8], MeOH [12], Figure 3b), the C_6_ homolog (*R*)-**9b** was about 30% less intense when measured in 2,2,2-trifluoroethanol (TFE, *A* = 119; Figure 3a), but essentially within the expected range.

### 2.3. Enantiomeric Purity of **1a**—Geographic Variation

**Figure 4:**
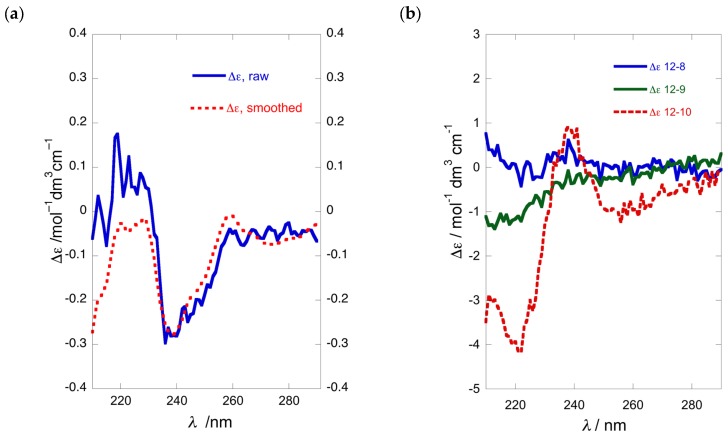
ECD of **1b** (MeOH, 23 °C): (**a**) ‘near racemic’ (*R*)-**1b** (blue) and fitted curve (red); (**b**) overlay of three samples of **1b** prepared from **1a** collected at different locations (Table 1).

We examined the optical purity of **1a** from various samples of *P. opacum* and one of *P. cereum*, collected respectively from Whangateau Harbor and Princes Island, North Island of New Zealand, by conversion to the *N*,*O*-dibenzoate **1b**, and measurement of their ECD spectra and calculation of %ee by comparison with CE data observed for model compound **4b** (Table 1). To our surprise, a re-collection of *P. opacum* from the same location as reported previously [7] (Entry 4), which revealed that unlike the original collection, the newer sample of **1a** was nearly racemic (Table 1, Entry 5; Figure 4a). It was noted that this new collection of *P. opacum* contained a mixture of two ascidian color-morphs; white and beige, which could possibly have some bearing on the configurational hetereogeneity of **1a.** Consequently, another collection of ascidian material was undertaken, taking care to separate the two color-morphs. Analysis of **1a** from these two color-morphs (entries 1 and 2; Figure 4b) revealed that both samples were also nearly racemic with %ee within experimental error. It appeared that none of the collections of *P. opacum* from that same location in the same season contained **1a** of high optical purity. Similar ECD analysis of the dibenzoyl derivative of **1a** isolated from specimens of *P. cereum* collected at Princes Island (Entry 3) identified the natural product as a scalemic mixture (~49%ee) of predominantly (*S*)-configuration (Figure 4b). 

To further investigate the enantiopurity of samples of **1a** we prepared CDA derivatives using the α-methoxyphenylacetic acid (MPA) method of Riguera [20]. EDC-promoted coupling of **1a** with either (*S*)- or (*R*)-MPA afforded *bis*-(*N*,*O*)-MPA derivatives **1c** and **1d**, respectively. 

In order to avoid possible introduction of bias to the ratio of diastereomeric products through fractionation during column chromatography, the crude CDA derivatization products were evaluated directly by ^1^H-NMR (see Supporting Information). While contaminant signals overlapped the ^1^H-NMR OMe resonances (δ_H_ 3.23–3.40), the CαH region (δ_H_ 4.40–4.80) was relatively un-obscured and so used for evaluation of enantiopurity. The MPA derivatives of synthetic (*S*)-**1a** prepared by Sun et al., identified CαH resonances at δ_H_ 4.80 (*S*-MPA ester), δ_H_ 4.71 (*R*-MPA ester), δ_H_ 4.59 (*R*-MPA amide), and δ_H_ 4.40 (*S*-MPA amide) (CDCl_3_) [10]. The MPA derivatives of synthetic (*S*)-**3a** displayed a single pair of CαH resonances (>99%ee; Figure S21, see Supplementary Material), and application of the configurational model [20] confirmed the AC (Table 1; Entry 7). In the case of **1a** sourced from *P. cereum*, ^1^H-NMR spectra of both MPA-derivatized samples **1c** and **1d** displayed the presence of all four CαH resonances, showing that **1a** (Figure S22) was a mixture of enantiomers favoring an excess of *S* (42%ee) when quantified using **1c**, or 26%ee for **1d** (average of 34%ee). The optical purities of **1a** samples obtained from other collections of *P. opacum* were evaluated in a similar manner (Entries 1, 2 and 5); in each case very low %ee values were observed (data not shown).

## 3. Discussion

### 3.1. Variable Acylation Efficiency of Long-Chain 1-AAs

The unexpected chain-length dependence of benzoylations or naphthoylations of 1-AAs under conventional conditions (e.g., Method A) suggests subtle differences in solution phase that amplify to larger differences in rates of reaction for the second reaction step: The acylation of the secondary OH group. It should be stressed that diacetylation of 1-AAs does not suffer these dramatic changes: Diacetylation reactions of long-chain 1-AAs under standard conditions (neat 1:1 pyridine-acetic anhydride as solvent and reactant) were unimpeded and high-yielding [12] as expected with the more reactive acylation reagent and larger over-equivalencies. We presume the uniform success of acylation of long-chain 1-AAs under the conditions of Method B avoids micelle formation when carried out in solvents of lower dielectric constant (e.g., CH_2_Cl_2_ or DCE) than pyridine.

### 3.2. The EC ECD Conformational Model is Upheld 

**Figure 5:**
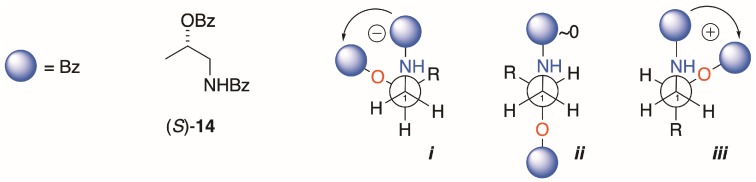
Model compound (*S*)-**14**, its most stable conformations (***i***–***iii***),and prediction of sign of the split-Cotton effect (CE, after Kawai et al. [21]. See also [8]; p. 159).

Interpretation of the EC ECD spectra of **1b** and **2b** are based on Nagai’s observations of the sign of the split CE and conformation analysis of (*S*)-**14**, the simplest chiral homolog of an *N*,*O*-dibenzoyl-1-AA (Figure 5) [21]. Of the three dominant gauche conformers, ***i*** is disfavored by steric hindrance. Conformer ***ii*** is expected to show Δ*ε* ~0 due to the antiperiplanar arrangement of the transition dipole moments of the two Bz chromophores, leaving ***iii*** as the favored conformer with the positive helicity inducing a negative split CE. The measured ECDs of the homologs **4b**, **9b** and **13a**,**b** in this study all conform to this fundamental model; *S* enantiomers correlate with positive split-CE and *R* enantiomers correlate with a negative split CE. The strongest CEs in the series are observed by replacement of the Bz chromophore with 2-naphthoyl group (*viz*. **9a**,**b** and **13a**,**b**; [12]); consequently, introduction of the latter chromophore is preferred for stereoanalysis of natural 1-AAs.

Finally, the naphthoyl-naphthimido derivatives, while well-served for ‘fingerprinting’ *threo* and *erythro* isomers of 2-amino-1,3-diols such as sphingosines and dihydrosphingosines [18,19], offer no advantage over a simple di-naphthoyl derivative (e.g., **9b** or **13a**,**b**) for assignments of 1-AAs; the latter derivatives offers simplicity, exceptional sensitivity combined with ease of preparation and interpretation of their ECD spectra.

### 3.3. Semi-Quantitative Comparison of ECD and CD methods for Stereoassignment of 1-AAs

The value of %ee, defined by a ratio (Equation (1)) of the sum and difference of concentrations, *C*, of (+)- and (−)-enantiomers, is prone to errors of measurement in each. Comparisons of [α]_D_ for sample and standard have long been used for determinations of %ee, but they become flawed when the magnitudes of the specific rotations are low, or when sample solutions of different concentrations induce non-linearity. The complementarity of the two methods used in this work—ECD and CDA derivatization with MPA—for establishing %ee and AC of 1-AAs, such as **1a**, is apparent from a simple semi-quantitative analysis of the sources of error and observation of the discrepancies between the two methods (Table 1). The ECD method is best suited for %ee of near-racemic samples where the null point corresponds to a racemate (∆*ε*_sample_/∆*ε*_standard_ = 0). The sign and magnitude of non-zero split-CE signal (~4%ee and higher) indicate configuration and non-racemic mixtures, respectively, with errors in the latter associated only with absolute *A* values and the signal-to-noise of the measurement.

(1) $$\%\mathrm{ee}=\frac{\left[C\left(+\right)-C\left(-\right)\right]\times 100}{\left[C\left(+\right)+C\left(-\right)\right]}=\frac{{\left[\alpha \right]}_{D\;\mathrm{sample}}\times 100}{{\left[\alpha \right]}_{D\;\mathrm{standard}}}=\frac{{\Delta \varepsilon }_{\mathrm{sample}}\times 100}{{\Delta \varepsilon }_{\mathrm{standard}}}$$

EC ECD of vicinal *N*,*O*-di-arenoyl derivatives has two advantages over [α]_D_ for determination of %ee: Δ*ε* is a molar quantity that conforms to the Beer–Lambert law and the absolute magnitude of the Δ*ε* signal (or *A*) is typically larger. Consequently, EC ECD is preferred for AC determination and quantitation of %ee, but there are limitations. Errors in %ee will be larger for samples near 100%ee where the ratio is obtained from Δ*ε*_sample_ and Δ*ε*_standard_ values that differ little in magnitude. MPA-derivatization—although requiring the preparation of separate *S*- and *R*- derivatives—is equally reliable for assigning AC of **1a**. For optical purity, however, the MPA method is better suited for measurements within a different range of %ee values (closer to 50%ee) where cumulative bias in integral measurement (*C* (+) or *C* (−)) due to instrumental factors (e.g., absolute error of integrals, partial NMR signal saturation, poor S/N and non-linear error propagation) is lowest. 

### 3.4. Geographic Variation of **1a**

**Figure 6:**
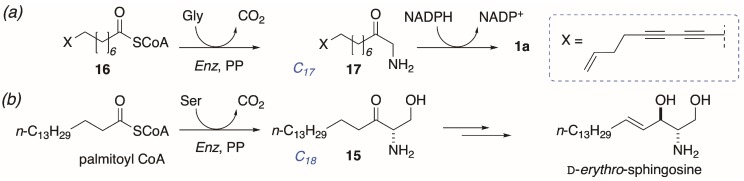
Hypothetical origin of (**a**) distaminolyne A (**1a**) modelled on (**b**) the known biosynthesis of d-*erythro*-sphingosine ([1]; p. 1622) and sphinganine-mycotoxins [22]. PP = pyridoxal 5′-phosphate.

The finding of heterogeneous enantiomeric compositions of different samples of **1a** is unusual, and suggestive of a biosynthesis from lipid and amino acid precursors that differs from conventional long-chain base biosynthesis at one or more later steps. The proposed biosyntheses of 1-AA natural products (Figure 6a) is modeled on sphinganine-mycotoxin biosynthesis [22] with parallels to mammalian sphingosine biosynthesis (Figure 6b) [1]: Condensation of the C_16_ palmitoyl CoA ester with Ser, with concomitant decarboxylation, gives ketosphingosine (**15**) that undergoes stereospecific reduction of the keto group by NADPH and subsequent oxidative desaturation (Strictly, desaturation occurs on the corresponding ceramide, and free d-*erythro*-sphingosine is released through the action of a ceramidase ([1] p. 1622])) to give (2*S*,3*R*)-sphingosine. 

The latter sequence is highly conserved in higher animals: All mammalian sphingosines, including variants with different chain-lengths, are of the d-configuration. By comparison, the proposed biosynthesis of **1a** would appear to proceed by activation of a hypothetical long-chain diyne-ene C_17_ fatty acid CoA ester **16**, followed by decarboxylative condensation with Gly instead of Ser, and reduction of the resultant ketone **17** by NADPH. Unlike **15**, the proposed intermediate aminoketone **17** undergoes reduction with lower stereofidelity than reduction of **15**. In contrast, all natural 2-AAs appear to be biosynthesized with hallmark high stereofidelity even when the relative and absolute configurations differ from species to species [23]. 

### 3.5. Broader Implications

The foregoing results should have broader applications to both stereochemical analysis of other aminoalkanols from Nature and synthesis. While reliable, prior analyses of 2-AAs exploiting ECD of *bis*-*N*,*O*-dibenzoyl derivatives (e.g., halisphingosines; [15]) suffered the same problem of lower-than desirable yields. The corresponding *bis*-*N*,*O*-di-(2-naphthoyl) derivatives, prepared by Method B, should ameliorate problems of compound yield and even permit sub-nanomole analyses (e.g., *Method C*) on vanishingly small sample sizes. In the asymmetric syntheses of 1-AAs and 2-AAs, the challenges of determination of %ee can better be met by ECD, or CDA derivatization and NMR; methods that nicely overcome the limitations imposed by sole reliance on comparisons of [α]_D_.

## 4. Materials and Methods

All UV-vis and chiroptical measurements were made with solutions prepared in spectroscopic grade solvents; MeOH, CHCl_3_ (stabilized with amylenes) or CF_3_CH_2_OH (TFE), purchased from Acros Organics (NJ, USA). Optical rotations were recorded on a JASCO P2000 digital polarimeter (Easton, MD, USA) at the D-line of Na emission on solutions in quartz cells (0.100 dm pathlength). UV-visible spectra were recorded on a JASCO V630 dual beam spectrometer (Easton, MD, USA) in quartz cuvettes (1.00 cm or 0.100 cm pathlength). Electronic circular dichroism spectra were recorded on either a Jasco J810 spectropolarimeter (Easton, MD, USA) or an Applied PhotoPhysics Chirascan with solutions in quartz cells (0.100 or 0.500 cm). HRMS were acquired on two instruments: a Bruker micrOTOF Q II mass spectrometer (Billerica, MA, USA) using external calibration of sodium formate clusters, and an Agilent 6230 TOFMS (Santa Clara, CA, USA) with a Jetstream electrospray ionization source, calibrated by using Agilent ESL-L Low Concentration Tuning Mix (Part number G1969-85000, Agilent Technologies). ^1^H-NMR spectra were recorded at 500.16 MHz on a Jeol ECA 500 spectrometer, at 399.91 MHz on a Mercury 400, at 400.13 MHz on a Bruker Avance AVIII 400 MHz or at 500.19 MHz on a Bruker Avance AVIII-HD 500 MHz spectrometer (Billerica, MA, USA). ^13^C-NMR spectra were recorded at 125.69 MHz on a Varian VX 500 spectrometer (Palo Alto, CA, USA) equipped with an Xsens ^13^C{^1^H} cryoprobe. Spectra were referenced to solvent (residual CHCl_3_ *δ* 7.26 ppm; CDCl_3_, *δ* 77.16 ppm; residual CHD_2_OD *δ* 3.31 ppm; CD_3_OD *δ* 49.00 ppm). Dry pyridine was distilled from CaH_2_ under an atmosphere of N_2_, and dry CH_2_Cl_2_, CH_3_CN were obtained by passage through commercial basic alumina cartridges under an atmosphere of Ar. Jacobsen’s catalysts [24] (*S*,*S*)-(+)- and (*R*,*R*)-(−)-*N*,*N′*-*bis*(3,5-*tert*-butylsalicylidene)-2,2cyclohexanediaminecobalt(II), (±)-1,2-epoxybutane and (±)-1,2-epoxyhexane were obtained from Sigma-Aldrich (Milwaukee, WI, USA).

Animal material. The ascidian consisting of mixed color-morphs of *Pseudodistoma opacum* was collected on September 29, 2017, at a depth of 4–6 m in Whangateau Harbour entrance (36.3196° S, 174.7831° E), Northland, New Zealand and kept frozen until used. The white and beige color-morphs were collected separately from the same location on November 14, 2017 and kept frozen until used. A voucher specimen of the ascidian is held at NIWA, Wellington, New Zealand as MNP 99203. The organisms were collected under MPI/UoA special permit 549. The sample of *P. cereum* was collected on November 24, 2002, at a depth of 15 m from Princes Island (34.1786° S, 172.0418° E), Three Kings island group, Northland, New Zealand and kept frozen until used. A voucher specimen of *P. cereum* is held at NIWA as MNP7042.

Isolation and purification. The freeze-dried ascidians were macerated and cold extracted with MeOH (4 × 200 mL), and the extract filtered, concentrated in vacuo affording a green solid, which was subjected to repeated C_18_ reversed-phase column chromatography using a gradient of H_2_O (0.05% TFA) to 100% MeOH. Distaminolyne A∙TFA salt eluted with 70% MeOH/H_2_O as a pale yellow gum.

*Distaminolyne A* (NP-9-161-3, *P. opacum* mixed colors; **1a**): 30 mg, 0.08% dry wt; ${\left[\alpha \right]}_{D}^{20}$ +1.0 (*c* 1.0 MeOH); The ^1^H NMR spectrum (CD_3_OD, 500 MHz) was identical with the published data [7]; (+)-HRESIMS *m*/*z* 262.2163 [M + H]^+^, (calcd. for C_17_H_28_NO, 262.2165).

*Distaminolyne A* (NP-12-8-1, *P. opacum* white color-morph; **1a**): 27 mg, 0.2% dry wt; ${\left[\alpha \right]}_{D}^{20}$ +1.9 (*c* 0.73, MeOH); The ^1^H NMR (CD_3_OD, 500 MHz) spectrum was identical with the published data [7]; (+)-HRESIMS *m*/*z* 262.2161 [M + H]^+^, (calcd. for C_17_H_28_NO, 262.2165).

*Distaminolyne A* (NP12-9-1, *P. opacum* beige color-morph; **1a**): 7 mg, 0.1% dry wt; ${\left[\alpha \right]}_{D}^{20}$ −1.1 (*c* 0.70, MeOH); The ^1^H NMR (CD_3_OD, 500 MHz) spectrum was identical with the published data [7]; (+)-HRESIMS *m*/*z* 262.2159 [M + H]^+^, (calcd. for C_17_H_28_NO, 262.2165).

*Distaminolyne A* (NP-12-10-1, *P. cereum*; **1a**): 8.5 mg, 0.04% dry wt; ${\left[\alpha \right]}_{D}^{20}$ +1.5 (*c* 0.275, MeOH); ^1^H NMR (CD_3_OD, 500 MHz) δ 5.89–5.83 (1H, m), 5.12–5.08 (1H, m), 5.06–5.03 (1H, m), 3.76–3.73 (1H, m), 3.03 (1H, dd, *J* = 12.8, 3.0 Hz), 2.77 (1H, dd, *J* = 12.8, 9.5 Hz), 2.35 (2H, t, *J* = 6.6 Hz), 2.28–2.24 (4H, m), 1.56–1.29 (12H, m); ^13^C NMR (CD_3_OD, 125 MHz) δ 137.8, 116.3, 78.0, 77.1, 68.7, 66.7, 66.3, 46.1, 36.0, 33.7, 30.5, 30.1, 29.8, 29.5, 26.3, 19.6; (+)-HRESIMS *m*/*z* 262.2157 [M + H]^+^, (calcd. for C_17_H_28_NO, 262.2165).

*N,O-Dibenzoyl derivative***1b**: Distaminolyne A **1a** (NP-12-8-1) (3.7 mg, 14 µmol) was dibenzoylated with benzoic acid according to *Method B* to obtain, after RP C_18_ chromatography (1:9 water/methanol) dibenzoyl derivative **1b**, 2.6 mg (40%). ${\left[\alpha \right]}_{D}^{20}$ 0 (*c* 0.26, MeOH); UV (MeOH) λ_max_ (log ε) 203 (4.28), 228 (4.19), 267 (3.55), 282 (3.44); The ^1^H NMR (CD_3_OD, 500 MHz) spectrum was identical with the published data [7]; (+)-HRESIMS *m*/*z* 492.2500 [M + Na]^+^, (calcd. for C_31_H_35_NNaO_3_, 492.2509).

*N,O-Dibenzoyl derivative***1b**: Distaminolyne A **1a** (NP-12-9-1) (2.5 mg, 10 µmol) was dibenzoylated with benzoic acid according to *Method B* to obtain, after RP C_18_ chromatography (1:9 water/methanol) dibenzoyl derivative **1b**, 1.8 mg (40%). ${\left[\alpha \right]}_{D}^{20}$ −1.1 (*c* 0.18, MeOH); UV (MeOH) λ_max_ (log ε) 204 (4.05), 228 (3.98), 266 (3.31), 281 (3.17); The ^1^H NMR (CD_3_OD, 400 MHz) spectrum was identical with the published data [7]; (+)-HRESIMS *m*/*z* 492.2504 [M + Na]^+^, (calcd. for C_31_H_35_NNaO_3_, 492.2509).

*N,O-Dibenzoyl derivative***1b**: Distaminolyne A **1a** (NP-12-10-1) (3.5 mg, 13 µmol) was dibenzoylated with benzoic acid according to *Method B* to obtain, after RP C_18_ chromatography (1:9 water/methanol) dibenzoyl derivative **1b**, 2.6 mg (41%). ${\left[\alpha \right]}_{D}^{20}$ +0.4 (*c* 0.26, MeOH); UV (MeOH) λ_max_ (log ε) 205 (4.34), 226 (4.23), 267 (3.70), 282 (3.59); The ^1^H NMR (CD_3_OD, 400 MHz) spectrum was identical with the published data [7]; (+)-HRESIMS *m*/*z* 492.2504 [M + Na]^+^, (calcd. for C_31_H_35_NNaO_3_, 492.2509).

(*S*)-*MPA derivative* **1c**: Distaminolyne A **1a** (NP-9-161-3) (2.0 mg, 8 µmol) was treated with (*S*)-MPA according to *Method B* to yield 3.4 mg (80%) of crude product. ^1^H NMR (CDCl_3_, 500 MHz) diagnostic Cα*H* resonances of *bis*-MPA: δ 4.79 (0.96H, s), 4.71 (1H, s), 4.59 (1H, s), 4.41 (0.96H, s).

(*R*)-*MPA derivative* **1d**: Distaminolyne A **1a** (NP-9-161-3) (2.0 mg, 8 µmol) was treated with (*R*)-MPA according to *Method B* to yield 2.4 mg (56%) of crude product. ^1^H NMR (CDCl_3_, 500 MHz) diagnostic Cα*H* resonances of *bis*-MPA: δ 4.79 (0.81H, s), 4.71 (1H, s), 4.59 (1H, s), 4.41 (0.81H, s).

(*S*)-*MPA derivative* **1c**: Distaminolyne A **1a** (NP-12-8-1) (2.0 mg, 6 µmol) was treated with (*S*)-MPA according to *Method B* to yield 3.6 mg (84%) of crude product. ^1^H NMR (CDCl_3_, 400 MHz) diagnostic Cα*H* resonances of *bis*-MPA: δ 4.79 (1H, s), 4.71 (0.93H, s), 4.59 (0.93H, s), 4.41 (1H, s).

(*R*)-*MPA derivative* **1d**: Distaminolyne A **1a** (NP-12-8-1) (2.0 mg, 6 µmol) was treated with (*R*)-MPA according to *Method B* to yield 3.8 mg (89%) of crude product. ^1^H NMR (CDCl_3_, 400 MHz) diagnostic Cα*H* resonances of *bis*-MPA: δ 4.79 (0.68H, s), 4.71 (1H, s), 4.59 (1H, s), 4.41 (0.68H, s).

(*S*)-*MPA derivative* **1c**: Distaminolyne A **1a** (NP-12-9-1) (2.0 mg, 6 µmol) was treated with (*S*)-MPA according to *Method B* to yield 2.3 mg (54%) of crude product. ^1^H NMR (CDCl_3_, 400 MHz) diagnostic Cα*H* resonances of *bis*-MPA: δ 4.79 (0.97H, s), 4.71 (1H, s), 4.59 (1H, s), 4.40 (0.97H, s).

(*R*)-*MPA derivative* **1d**: Distaminolyne A **1a** (NP-12-9-1) (2.0 mg, 6 µmol) was treated with (*R*)-MPA according to *Method B* to yield 3.5 mg (82%) of crude product. ^1^H NMR (CDCl_3_, 400 MHz) diagnostic Cα*H* resonances of *bis*-MPA: δ 4.79 (0.51H, s), 4.71 (1H, s), 4.59 (1H, s), 4.41 (0.51H, s).

(*S*)-*MPA derivative* **1c**: Distaminolyne A **1a** (NP-12-10-1) (1.5 mg, 6 µmol) was treated with (*S*)-MPA according to *Method B* to yield 2.4 mg (75%) of crude product. ^1^H NMR (CDCl_3_, 500 MHz) diagnostic Cα*H* resonances of *bis*-MPA: δ 4.79 (1H, s), 4.71 (0.59H, s), 4.60 (0.59H, s), 4.40 (1H, s).

(*R*)-*MPA derivative* **1d**: Distaminolyne A **1a** (NP-12-10-1) (1.5 mg, 6 µmol) was treated with (*R*)-MPA according to *Method B* to yield 2.8 mg (88%) of crude product. ^1^H NMR (CDCl_3_, 500 MHz) diagnostic Cα*H* resonances of *bis*-MPA: δ 4.79 (0.4H, s), 4.71 (1H, s), 4.60 (1H, s), 4.40 (0.4H, s).

*(R)-1-Amino-2-hexanol,***5b**: 1-Amino-2-alkanols were prepared from the corresponding optically enriched 1,2-epoxides (>97%ee, prepared by Jacobsen’s hydrolytic kinetic resolution of the corresponding racemic epoxide [24] with (*S*,*S*)- or (*R*,*R*)-catalyst) by ammoniolysis using a variation of the published procedure for **4a**,**b** [12]. To an ice-cold, stirred solution of (*R*)-1,2-epoxyhexane (82.7 mg, 0.83 mmol) in EtOH (3.0 mL) contained in a 20 mL vial was added, dropwise, aqueous NH_4_OH (20% *v*/*v*, 3.0 mL). The vial was sealed tightly and heated at 60 °C for 23 h. The cooled contents of the vial were transferred to a round bottom flask and the volatiles removed under reduced pressure. The residue was taken up in CHCl_3_ and passed through plug of anhydrous Na_2_SO_4_ to give, after removal of solvent, amino alcohol (*R*)-**5b** as a straw yellow oil (210 mg, 58%). Distillation (twice, Kügelrohr, 120–30 °C/1 mm Hg) gave an analytically pure sample. ^1^H and ^13^C NMR and MS data for (*R*)-**5b** matched the literature values [25].

*(S)-1-Amino-2-butanol,***6a**: The title compound was prepared from (*S*)-1,2-epoxybutane according to the above-described procedure for **5b**, with final purification by distillation (Kügelrohr, 130 °C/1 mm Hg) have an analytically pure sample. Colorless oil, ${\left[\alpha \right]}_{D}^{20}$ +17 (*c* 1.0, CHCl_3_), lit. +7.3 (*c* 1.0, CHCl_3_) [26]; ^1^H NMR and ^13^C NMR matched the literature values [26].

Acylation, Method A: A solution of 1-amino-2-alkanol (1-AA, 1 equiv), freshly distilled benzoyl chloride (4 equiv) and DMAP (1 crystal) in pyridine (1-AA concentration ~0.1 M) was heated at 60 °C under an atmosphere of N_2_ for 24–48 h. The volatiles were removed under high vacuum and the residue purified by elution (CH_2_Cl_2_) through a plug of basic alumina, followed by flash chromatography (silica or RP C_18_) followed by HPLC (silica or RP C_18_) if required.

Acylation, Method B: A solution of benzoic or 2-naphthoic acid (~1 mmol, 5 equiv), EDC∙HCl (5.5 equiv) and DMAP in CH_2_Cl_2_ was stirred at 0 °C for 20 min under N_2_. A solution of 1-AA in CH_2_Cl_2_ was (final concentration of 1-AA ~0.01 M) and the mixture warmed to rt over 24 h. Additional CH_2_Cl_2_ (20 mL) was added and the mixture washed successively with equal volumes of 10% HCl, water, NaHCO_3_ (satd.) and H_2_O. After drying (Na_2_SO_4_), the volatiles were removed and the residue purified by flash chromatography (silica), followed by HPLC (silica or RP C_18_) if required. 

Acylation, Method C: A standard solution was prepared by dissolving EDC∙HCl (18.6 mg, 97 µmol), DMAP (11.2 mg, 97 µmol) and 2-naphthoic acid (16.3 mg, 95 µmol) in 1,2-dichloroethane (DCE, 820 µL; final concentration 0.13 M in 2-naphthoic acid). A solution of 1-AA in DCE (6.1 µg, 7.5 µL, 0.35 µmol) and an aliquot of the standard solution (7.5 µL, 0.98 µmol, 2.8 equiv.) were transferred into a melting point capillary using a gas tight syringe which was then flame sealed. The capillary tube was placed into a melting point apparatus set at 68 °C and left for 60 min, after which the liquid contents of the tube were removed with a narrow capillary and applied directly onto a glass backed TLC plate (6 × 6 cm, silica, 250 µm, prewashed by 2× development in 1:1 EtOAc/*n*-hexane) in replicate lanes (*n* = 6). After development of the TLC plate in 1:4 EtOAc/*n*-hexane, the fluorescent spots corresponding to the *N*,*O*-dinaphthoyl-1-AA product (for (*R*)-**12b**, *R*_f_ 0.20) were scraped from the plate into a 6 mL vial containing a magnetic stir bar, and the mixture extracted with TFE (1.0 mL) by vigorous stirring for 30 min. The vial was centrifuged and measurements of the UV-vis and ECD spectra were carried out directly on the supernatant (30–40% yield based on absorbance at *λ*_max_ 231 nm). 

*N,O-Dibenzoyl-1-amino-2-decanol (R)-***4b**: (*R*)-1-Amino-2-decanol (**3b**) (2.5 mg, 14 µmol) was dibenzoylated with benzoic acid according to *Method B* to obtain, after ‘pencil column’ chromatography (silica, 1:3 EtOAc/*n*-hexane) dibenzoyl derivative (*R*)-**4b** (7.3 mg, quant). ${\left[\alpha \right]}_{D}^{20}$ −24.1 (*c* 1.48, MeOH). lit −26.2 (*c* 1.78, MeOH) [12]. The ^1^H-NMR spectrum (CDCl_3_) was identical with the published data [12].

*Naphthoylation of Amino Alcohol (R)-***5b**: 2-Naphthoyl chloride (304 mg, 1.60 mmol, 2.5 equiv), freshly prepared from 2-naphthoic acid (SOCl_2_, reflux), was added to a stirred solution of 1-AA (*R*)-**5b** (75.3 mg, 0.64 mmol) and DMAP (1 crystal) in pyridine (5.0 mL) under N_2_, and the mixture heated at 60 °C for 96 h. The volatiles removed under high vacuum and the pale pink semi-crystalline residue (220.4 mg) was eluted through a plug of basic alumina with CH_2_Cl_2_. The residue obtained by removal of solvent was separated by flash chromatograph (2:98 MeOH/CH_2_Cl_2_). The UV-absorbing fractions (TLC) were combined and, after removal of solvent, the residue was triturated with warm diethyl ether to give white crystals of mono-naphthamide (*R*)-**7b** (29.5 mg, 18%). The supernatant was concentrated and the residue (4.3 mg) separated, first on a ‘pencil column’ (1% MeOH/CH_2_Cl_2_) followed by HPLC (silica, 5 µ, 40:60 EtOAc/*n*-hexane, 3.0 mL∙min^−1^) to give dinaphthoyl derivative (*R*)-**9b** (1.2 mg, 0.5%) and oxazoline **8** (0.6 mg, 0.4%). With *Method B*, (*R*)-**5b** (3.2 mg, 19 µmol) was converted into (*R*)-**9b** (4.2 mg, 54% yield) after purification by pipette column (silica, 20% EtOAc-hexane).

*Mono-Naphthamide***7b**: Recrystallized from CDCl_3_, m.p. 135 °C; ${\left[\alpha \right]}_{D}^{23}$ −5.7 (*c* 0.98, MeOH); ^1^H NMR (CDCl_3_, 500 MHz) δ 8.29 (s, 1H, H-1′), 7.88–7.81 (m, 4H), 6.91 (bt, 1H, NH), 3.85 (m, 1H, H-2), 3.76 (ddd, 1H, *J* = 13.5, 7.0, 3.0 Hz, H-1a), 3.45 (ddd, 1H, *J* = 13.5, 8.0, 5.0 Hz, H-1b), 2.84 (bs, 1H, OH), 1.53 (m, 2H), 1.44 (m, 1H), 1.34 (3H), 0.90 (t, 3H, *J* = 7.0 Hz, H_3_-6). ^13^C NMR (CDCl_3_, * = interchangeable) 168.6 (C, C=O), 134.9 (C, C-2′*), 132.7 (C, C-4a*), 131.5 (C, C8a*), 129.0 (CH), 128.6 (CH), 127.85 (CH), 127.82 (CH), 127.7 (CH), 126.9 (CH), 123.7 (CH), 71.6 (CH, C-2), 46.4 (CH_2_, C-1), 35.0 (CH_2_, C-3), 27.8 (CH_2_, C-4), 22.8 (CH_2_, C-5), 14.2 (CH_2_, C-6); ESI TOF HRMS *m*/*z* 294.1468 [M + Na]^+^ calcd. for C_17_H_21_NO_2_Na^+^ 294.1465.

*Oxazoline***8**: ${\left[\alpha \right]}_{D}^{23}$ +2.6 (*c* 0.50, CHCl_3_); ^1^H NMR (CDCl_3_, 500 MHz) δ 8.43 (bd, 1H, *J* = 1.5 Hz, H-1′), 8.03 (dd, 1H, *J* = 8.5, 1.5 Hz, H-3′), 7.92 (d, 1H, *J* = 7.5 Hz, H-8′), 7.87–7.86 (m, 2H), 7.57-7.7.50 (m, 2H); ESI TOF HRMS *m*/*z* 254.1539 [M+H]^+^, calcd. for C_17_H_20_NO^+^ 254.1539.

*N,O-di-(2-Naphthoyl)-1-amino-2-hexanol (R)-***9b**: Colorless oil, ${\left[\alpha \right]}_{D}^{23}$ −67 (*c* 0.8, TFE); ECD (TFE) δ 228 (∆ε +53), 236 (0), 244 (−66); ^1^H NMR (CDCl_3_, 500 MHz) δ 8.65 (s, 1H, H-1′), 8.27 (s, 1H, H-1″), 8.09 (d, 1H, *J* = 8.5 Hz, H-3′), 7.97 (d, 1H, *J* = 8.0 Hz, H-3″), 7.91 (m, 6H), 7.79 (d, 1H, *J* = 8.5 Hz), 7.62-7.50 (m, 4H), 6.92 (bt, 1H, *J* = 5.0 Hz, NH), 5.42 (m, 1H, H-2), 3.91 (ddd, 1H, *J* = 14.5, 5.0, 3.5 Hz, H-1a), 3.85 (ddd, 1H, *J* = 14.5, 9.0, 5.0 Hz, H-1b), 1.92 (m, 1H, H-3a), 1.84 (m, 1H, H-3b), 1.5 (m, 2H, H_2_-4), 1.42 (m, 2H, H_2_-5), 0.94 (t, 3H, *J* = 7.3 Hz, H_3_-6); ESI TOF HRMS *m*/*z* 426.2061 [M + H]^+^ calcd. for C_28_H_28_NO_3_^+^ 426.2064.

*Tribenzoyl-1-amino-butanol (S)-***10a***:* A solution of 1-AA (*S*)-**6a** (66.7 mg, 0.75 mmol) in dry pyridine (2.0 mg) and DMAP (1 crystal) was treated with freshly distilled benzoyl chloride (271 µL, 2.99 mmol) and the mixture heated at 60 °C under at atmosphere of N_2_ with stirring for 48 h. The volatiles were removed under high vacuum and the semi-crystalline residue (1.28 g) taken up in CH_2_Cl_2_ and filtered through a plug of basic alumina. The eluate was concentrated and the residue separated on a ‘pencil column’ (silica, elution with 1:4 EtOAc/*n*-hexane) to give a non-polar, clear oil (67 mg) that was further purified by HPLC (silica, 10 × 250 mm, 5 µ, 1:4 EtOAc/*n*-hexane) to give tribenzoyl derivative (*S*)-**10b** (14.9 mg, 5%). ${\left[\alpha \right]}_{D}^{22}$ +44.6 (*c* 2.92, MeOH); ^1^H NMR (CDCl_3_, 500 MHz) δ 7.81 (d, 2H, *J* = 7.5 Hz, H-2″,6″), 7.39 (t, 1H, *J* = 7.4 Hz, H-4″), 7.33 (d, 4H, *J* = 7.5 Hz, H-2′,6′), 7.23 (t, 2H, *J* = 7.5 Hz, H-3″,5″), 7.12 (t, 2H, *J* = 7.5 Hz, H-4′), 7.02 (t, 4H, *J* = 7.5 Hz, H-3′,5′), 5.65 (m, 1H, H-2), 4.53 (dd, 1H, *J* = 14.0, 9.1 Hz, H-1a), 4.25 (dd, 1H, *J* = 14.1, 2.8 Hz, H-1b), 1.87 (m, 2H, H_2_-3), 1.06 (t, 3H, *J* = 7.5 Hz, H_3_-6). ^13^C NMR (CDCl_3_) δ (C, N(C=O)_2_), 166.4 (C, O(C=O)), 136.3 (C, C_2_-1′), 132.8 (CH, C-4″), 131.9 (C, C_2_-4′), 130.0 (CH, C-1″), 129.6 (CH, C-2′,6′), 129.0 (CH, C_4_-2′,6′), 128.16 (CH, C_2_-3′), 128.15 (CH, C-3″), 75.0 (CH, C-2), 49.9 (CH_2_, C-1), 25.8 (CH_2_, C-3), 9.6 (CH_3_, C-4); HRMS *m*/*z* 424.1520 [M + Na]^+^, calcd. for C_25_H_23_NO_4_Na^+^ 424.1519.

*Naphthimide* (*R*)-**11b***:* A solution of the 1-AA (*R*)-**5b** (3.5 mg, 30.3 µmol) in dry pyridine (0.175 mL) was added to a stirred solution of freshly sublimed 2,3-naphthalenedicarboxylic acid anhydride (9.0 mg, 45.4 µmol, 1.5 equiv.) in dry pyridine (0.325 mL) and the mixture heated to 110 °C under N_2_ for 48 h. The volatiles were removed under a stream of N_2_. The residue was divided in half, and each applied to duplicate preparative TLC plates (silica, 200 × 200 × 0.25 mm). The plates were developed, twice (2:3 EtOAc/*n*-hexane) and the silica corresponding to the major fluorescent bands (TLC, *R*_f_ = 0.52, 1:1 EtOAc/*n*-hexane) were scrapped from the plates and extracted with EtOAc. The extracts were combined, filtered and the solvent removed from the filtrate to deliver (*R*)-**11b** (6.9 mg, 74%) as a colorless powder. ${\left[\alpha \right]}_{D}^{22}$ +7.0 (*c* 0.64, CHCl_3_); ^1^H NMR (CDCl_3_, 500 MHz) δ 8.35 (s, 2H, H-1′,4′), 8.05 (m, 2H, H-5′,6′), 7.71 (m, 2H, H-7′,8′), 3.94 (m, 1H, H-2), 3.88 (dd, 1H, *J* = 14.0, 3.5 Hz, H-1a), 3.82 (dd, 1H, *J* = 14.0, 8.0 Hz, H-1b), 2.46 (bs, 1H, OH), 1.58–1.48 (m, 2H, H_2_-3), 1.42–1.33 (m, 4H, H_2_-4, H_2_-5), 0.92 (t, 3H, *J* = 7.0 Hz, H_3_-6); ESI TOF HRMS *m*/*z* 320.1253 [M + Na]^+^, calcd. for C_18_H_19_NO_3_Na^+^ 320.1257.

*O-Naphthoyl-naphthimide* (*R*)-**12b**: An ice-cold solution of 2-naphthoic acid (4.0 mg, 32 µmol, 5.0 equiv.), DMAP (4.0 mg, 33 µmol, 5.1 equiv.) and EDC∙HCl (6.3 mg, 33 µmol, 5.1 equiv.) in CH_2_Cl_2_ (0.5 mL) was stirred for 30 min under an atmosphere of N_2_, then treated with a solution of naphthimide (*R*)-**11b** (2.2 mg, 6.4 µmol) in CH_2_Cl_2_ (0.3 mL). The solution was allowed to warm to 23 °C and stirred for 64 h, then diluted with additional CH_2_Cl_2_ (15 mL) and washed sequentially with 0.2M HCl, NaHCO_3_ (satd.) and water. After drying the solution (Na_2_SO_4_), the volatiles were removed and the residue (4.5 mg) purified by ‘pencil column’ chromatography (silica, 1:4 EtOAc/*n*-hexane) to give pure (*R*)-**12b** as a colorless oil (2.2 mg, 72%, *R*_f_ 0.20, 1:4 EtOAc/*n*-hexane). ${\left[\alpha \right]}_{D}^{22}$ −90 (*c* 0.44, TFE); ECD (TFE) δ 216 (∆ε +24.7), 240 (+25.8), 245 (0), 259 (−31.8); ^1^H NMR (CDCl_3_, 500 MHz, * = interchangeable) δ 8.55 (d, 1H, *J* = 1.4 Hz, H-1″), 8.29 (s, 2H, H_2_-1′,4′), 8.00 (m, 2H, H_2_-5″,8″), 7.98 (dd, 1H, *J* = 8.3, 1.4 Hz, H-3″), 7.94 (d, 1H, *J* = 8.0 Hz), 7.85 (d, 1H, *J* = 7.0 Hz), 7.83 (d, 1H, *J* = 8.5 Hz), 7.85 (dd, 1H, *J* = 7.7, 0.8 Hz), 7.83 (d, *J* = 8.3 Hz), 7.66 (m, 2H, H-6′,7′), 7.57 (td, 1H, *J* = 7.5, 1.5 Hz, H-6″*), 7.52 (td, 1H, *J* = 7.5, 1.0 Hz, H-7″*), 5.52 (m, 1H, H-2), 4.16 (dd, 1H, *J* = 14.0, 8.5 Hz, H-1a), 4.02 (dd, 1H, *J* = 14.0, 3.0 Hz, H-1b), 1.85 (m, 2H, H_2_-3), 1.49 (m, 2H, H_2_-4), 1.40 (m, 2H, H_2_-5), 0.92 (t*,* 3H, *J* = 7.3 Hz, H_3_-6); ESI TOF HRMS *m*/*z* 452.1847 [M + H]^+^, calcd. for C_29_H_26_NO_4_^+^ 452.1856.

*N,O-di-(2-Naphthoyl)-1-amino-2-decanol (S)-***13a**: A sample of the 1-AA (*S*)-**3a** (4.5 mg, 16 µmol) was acylated with 2-naphthoic acid (5 equiv.) using *Method B* to give, after standard workup and purification by pipette column (silica, 1:5 *n*-hexane/CH_2_Cl_2_), **13a** (3.5 mg, 46%). ${\left[\alpha \right]}_{D}^{20}$ +94 (*c*, 0.16, CHCl_3_)), which displayed ^1^H and ^13^C NMR spectra identical with those of (*R*)-**13b** [12]. ESI TOF HRMS *m*/*z* 504.2501 [M + Na]^+^, calcd. for C_32_H_35_NaNO_3_^+^ 504.2509.

## 5. Conclusions

An optimized approach to assignment of absolute configuration of 1-amino-2-alkanols (1-AAs), based on ECD, is presented which avoids problems with prior methods, and streamlines analyses of multiple samples. Comparison and contrast of two methods for chiroptical analysis of 1-AAs—ECD and CDA derivatization-^1^H-NMR—revealed complementary advantages. An optimized *N*,*O*-dinaphthoyl 1-AA derivative lends clarity of interpretation of ECD spectra with sufficient sensitivity for ‘sub-nanomole scale’ stereoassignments. Chiroptical investigations of multiple samples of the 1-AA, distaminolyne A (**1a**) extracted from *Pseudodistoma* spp., showed that configuration and enantiomeric purity varied with collections, a behavior that is unlike 2-AA natural products that, so far, have been found to be stereochemically homogenous. A promiscuous biosynthesis for **1a** is proposed to account for the difference.

## Figures and Tables

**Table 1:** Optical Purities of *N*,*O*-Dibenzoyl Derivatives of **1a**^1^–**3a** by ECD and CDA Methods.

Entry	Compounds	Specimen Code	*S/R* ECD	*A* ^2^	%ee ^2^ECD	%ee ^3^ (*S*)-MPA	%ee ^4^ (*R*)-MPA	%ee ^5^ MPA	Ref.
1	**1a**	NP-12-8-1		~0	~0	4 (*S*)	20 (*S*)	12 (*S*)	^6^
2	**1a**	NP-12-9-1		~0	~0	2 (*R*)	32 (*S*)	15 (*S*)	^6^
3	**1a** ^7^	NP-12-10-1	*S*	5	49	26 (*S*)	42 (*S*)	34 (*S*)	^6^
4	**1a**	JW-4-51-5	*S*	12.7	~100	-	-	-	^6^
5	**1a**	NP-9-161-3	*R*	0.33	2.9	2 (*R*)	10 (*S*)	4 (*S*)	^6^
6	**2a** ^8^	90-06-045	*R*	15.3	–	-	-	-	[9]
7	**4a**	–	*S*	10.9	>97 ^9^	>99 (*S*)	-	-	[12]
8	**4b**	–	*R*	11.4	>97 ^9^	-	-	-	[12]
									

^1^ Natural samples of **1a**, isolated from *Pseudodistoma opacum*, collected at Ti Point, North Island, New Zealand, were converted to *N*,*O*-dibenzoyl derivative **1b** by *Method B* as described in the Experimental. ^2^ See footnote on previous page for the definition of *A.*%ee ECD calculated from 100x*A*(**1b**)*/A*(**4b**). ^3^ (*S*)- and (*R*)-α-methoxyphenylacetic (MPA) derivatives prepared by Method B [12]. %ee calculated from integral ratio of Cα−H ^1^H-NMR resonances of *bis*-(*S*)-MPA derivative. ^4^ %ee calculated from integral ratio of Cα−H ^1^H-NMR resonances of *bis*-(*R*)-MPA derivative. ^5^ Average of (*S*)- and (*R*)-MPA %ee values. ^6^ This work. ^7^ From *P. cereum*. ^8^ **2a**, from an unidentified didemnid ascidian collected on the Great Barrier Reef, was converted to **2b** using a variant of Method A [9]. ^9^ Optical purity from (*S*)-1,2-epoxydecane (>97%ee), prepared from the Jacobsen’s hydrolytic kinetic resolution (HKR) reaction, and used in the synthesis of (*S*)-**4a**.

## Supplementary Materials

The following are available online. ^1^H and ^13^C-NMR spectra and HRMS spectra for (*R*)-**5b**, (*S*)-**6a**, (*R*)-**7b**, **8**, (*R*)-**9b**, (*S*)-**10a**, (*R*)-**11b**, (*R*)-**12b**, and (*S*)-**13a**, ^1^H-NMR spectra of MPA derivatives **1c**,**d** of partially of racemic **1a,** and MPA derivative of (*S*)-**13a**.
