# Supplementary material for: Enantiomeric Variability of Distaminolyne A. Refinement of ECD and NMR Methods for Determining Optical Purity of 1-Amino-2-Alkanols
Source: Molecules. 2018 Dec 27;24(1):90. doi: 10.3390/molecules24010090 (PMC6337674; doi:10.3390/molecules24010090)

Supplementary Materials

Enantiomeric Variability of Distaminolyne A. Refinement of ECD and NMR Methods for Determining Optical Purity of 1-Amino-2-Alkanols

A. Norrie Pearce,[†] Brent R. Copp[†] and Tadeusz F. Molinski*[,‡,§]

[†]*School of Chemical Sciences, University of Auckland, Private Bag 92019, Auckland 1142, New Zealand*
and
[‡]*Department of Chemistry and Biochemistry, and* [§]*Skaggs School of Pharmacy and Pharmaceutical Sciences, University of California, San Diego, 9500 Gilman Dr., La Jolla, CA 92093-0358, USA*

***** Correspondence: tmolinski@ucsd.edu; Tel.: +1-858-534-7115

Page	*Title*	*Content*
S1	Figure S1	^{1}H NMR Spectrum of (*R*)-**5a** (CD_3OD, 500 MHz)
S2	Figure S2	^{13}C NMR Spectrum of (*R*)-**5a** (CD_3OD, 125 MHz)
S3	Figure S3	^{1}H NMR Spectrum of (*S*)-**6a** ($CDCl_3$, 500 MHz)
S4	Figure S4	^{13}C NMR Spectrum of (*S*)-**6a** ($CDCl_3$, 125 MHz)
S5	Figure S5	^{1}H NMR Spectrum of (*R*)-**7b** ($CDCl_3$, 500 MHz)
S6	Figure S6	^{13}C NMR Spectrum of (*R*)-**7b** ($CDCl_3$, 125 MHz)
S7	Figure S7	^{1}H NMR Spectrum of **8** ($CDCl_3$, 500 MHz)
S8	Figure S8	^{1}H NMR Spectrum of (*R*)-**9b** ($CDCl_3$, 500 MHz)
S9	Figure S9	^{13}C NMR Spectrum of (*R*)-**9b** ($CDCl_3$, 125 MHz)
S10	Figure S10	^{1}H NMR Spectrum of (*S*)-**10a** ($CDCl_3$, 500 MHz)
S11	Figure S11	^{13}C NMR Spectrum of (*S*)-**10a** ($CDCl_3$, 125 MHz)
S12	Figure S12	^{1}H NMR Spectrum of (*R*)-**11b** ($CDCl_3$, 500 MHz)
S13	Figure S13	^{13}C NMR Spectrum of (*R*)-**11b** ($CDCl_3$, 150 MHz)
S14	Figure S14	^{1}H NMR Spectrum of (*R*)-**12b** ($CDCl_3$, 500 MHz)
S15	Figure S15	^{1}H NMR Spectrum of (*R*)-**12b** ($CDCl_3$, 500 MHz) - Expansions
S16	Figure S16	^{1}H NMR Spectrum of (*S*)-**13a** ($CDCl_3$, 400 MHz)
S17	Figure S17	ESI HRMS of (*R*)-**5b**, (*S*)-**6a** and (*R*)-**7b**
S18	Figure S18	ESI HRMS of **8**, (*R*)-**9b** and (*S*)-**10a**
S19	Figure S19	ESI HRMS of (a) (*R*)-**11b**, (b) (*R*)-**12b** and (c) (*S*)-**13a**
S20	Figure S20	^{1}H NMR Spectrum of *bis*-(*S*)-MPA derivative of (*S*)-**3a** ($CDCl_3$, 500 MHz)
S21	Figure S21	1H NMR Spectra of (a) bis (*S*)-MPA **1c** and (b) bis (*R*)-MPA **1d** highlighting CαH resonances and integrals ($CDCl_3$, 500 MHz)

Figure S1. ^{1}H NMR Spectrum of (*R*)-**5a** (CD_3OD, 500 MHz).

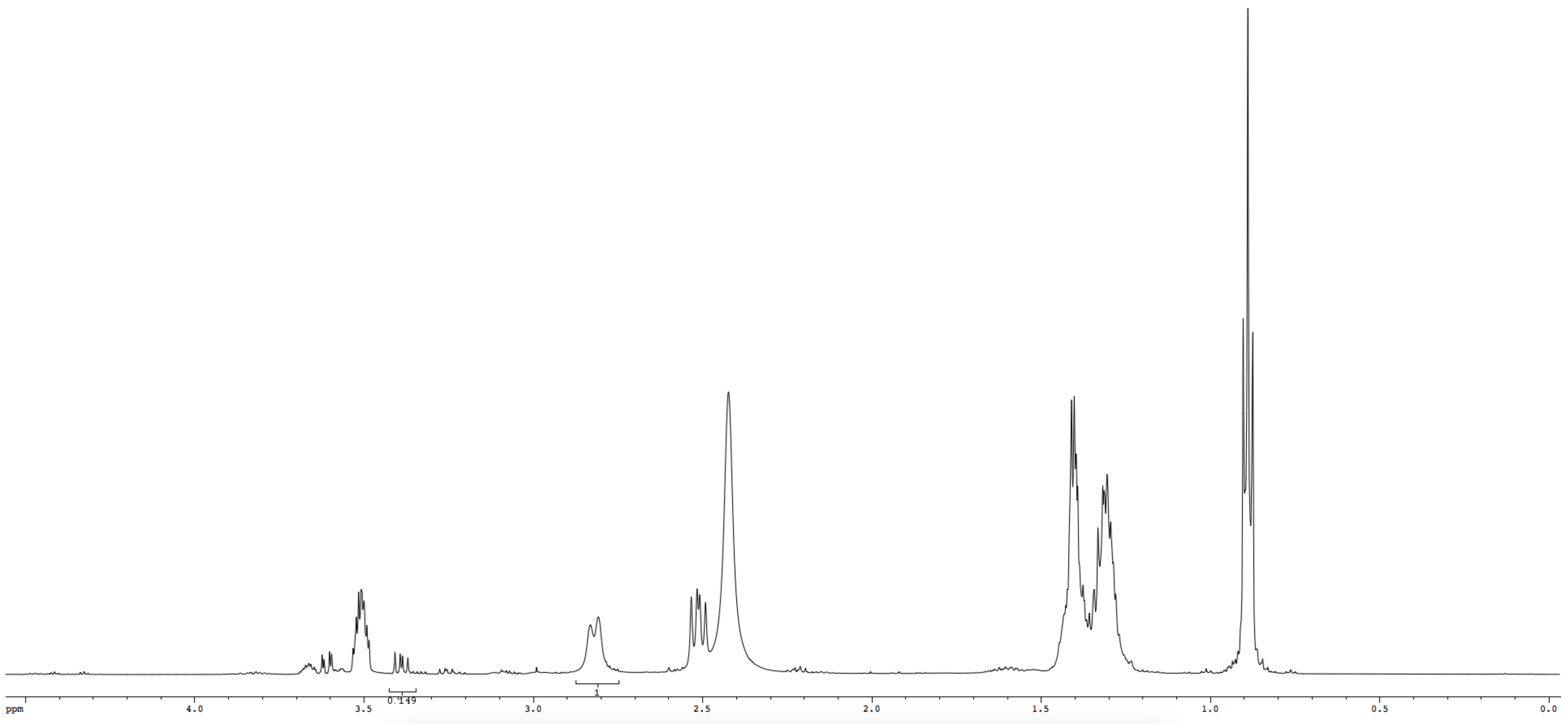

Figure S2. ^{13}C NMR Spectrum of (*R*)-**5a** (CD_3OD, 125 MHz).

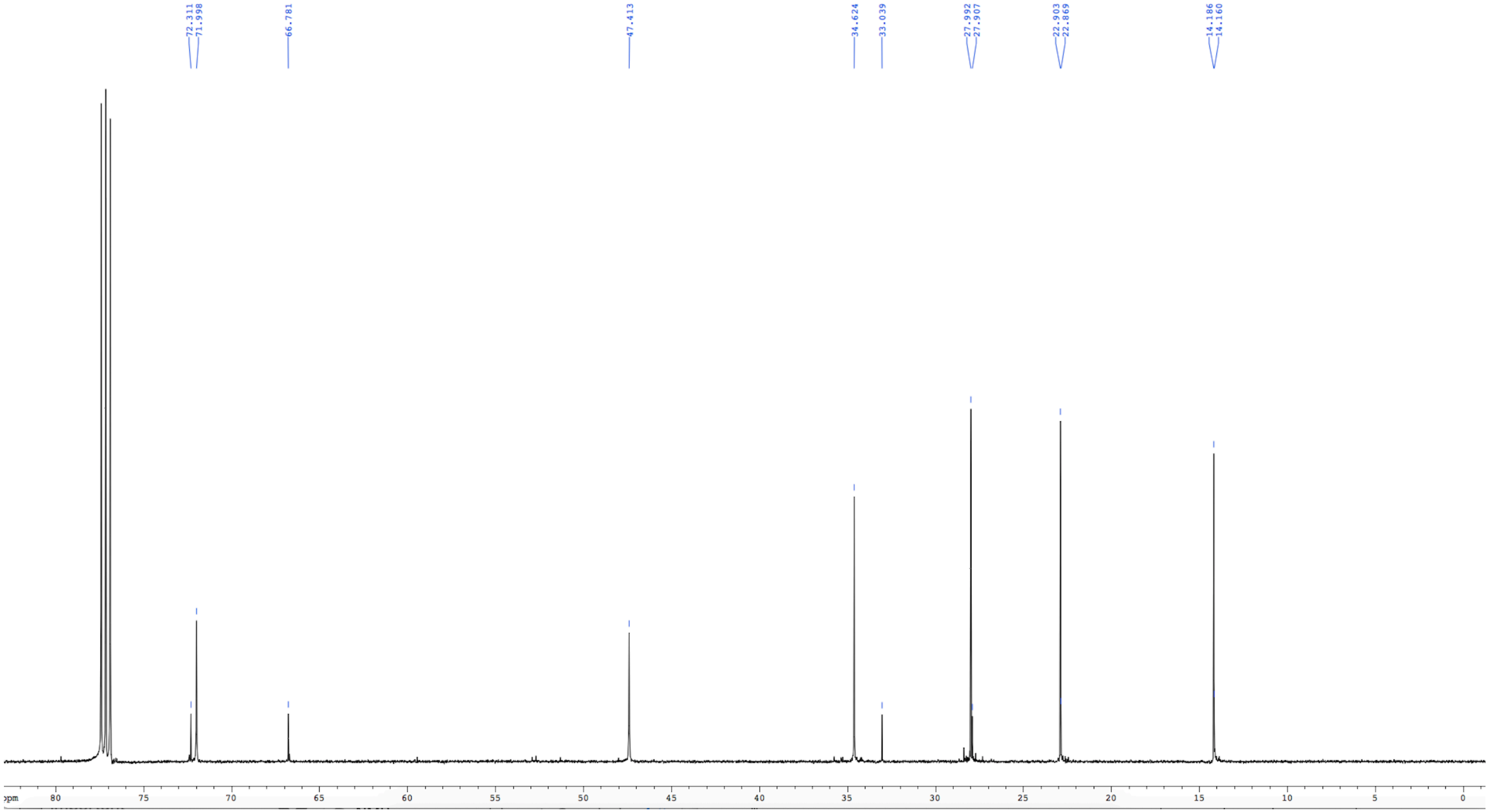

Figure S3. ^{1}H NMR Spectrum of (*S*)-**6a** ($CDCl_3$, 500 MHz).

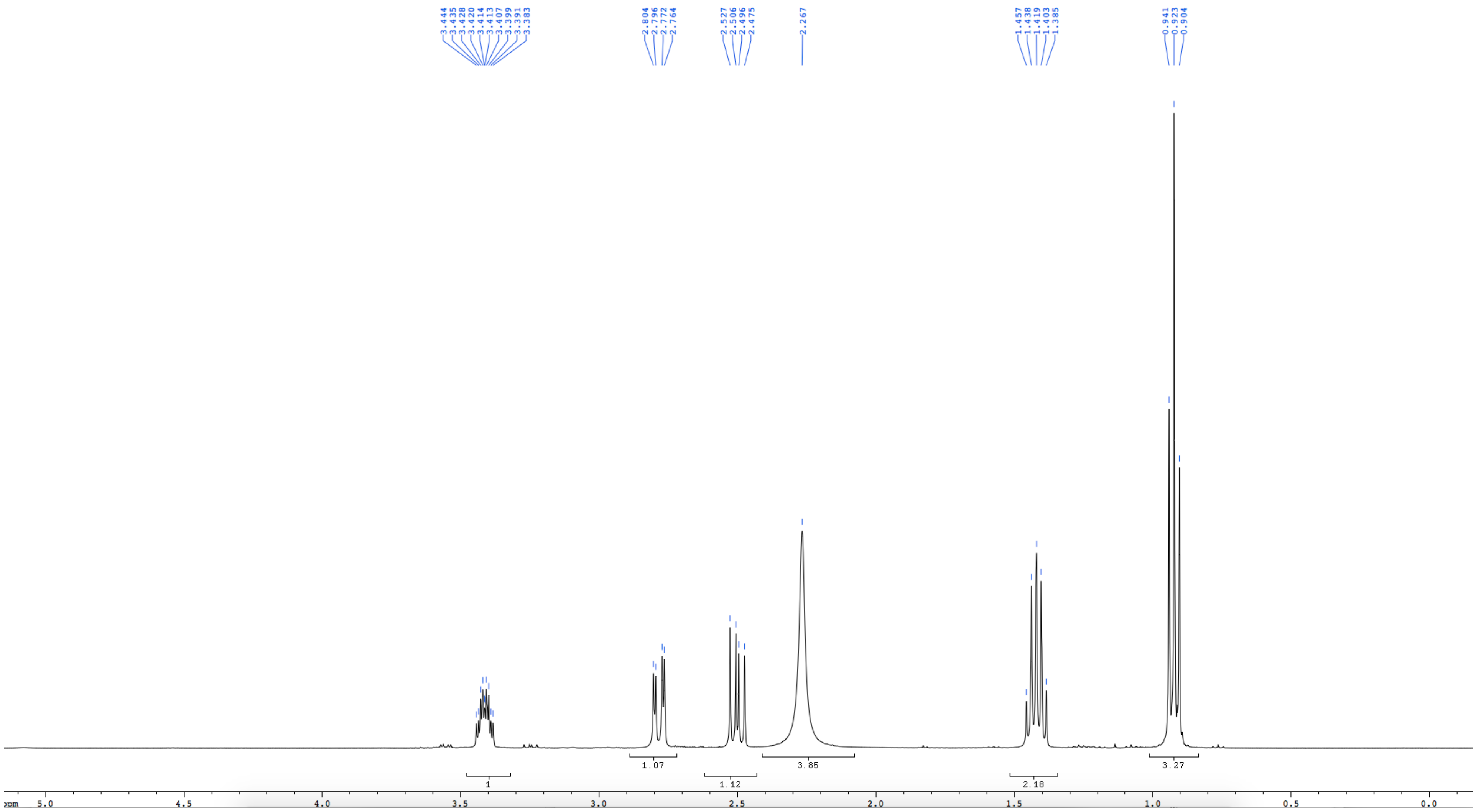

Figure S4. ^{13}C NMR Spectrum of (*S*)-**6a** ($CDCl_3$, 125 MHz)

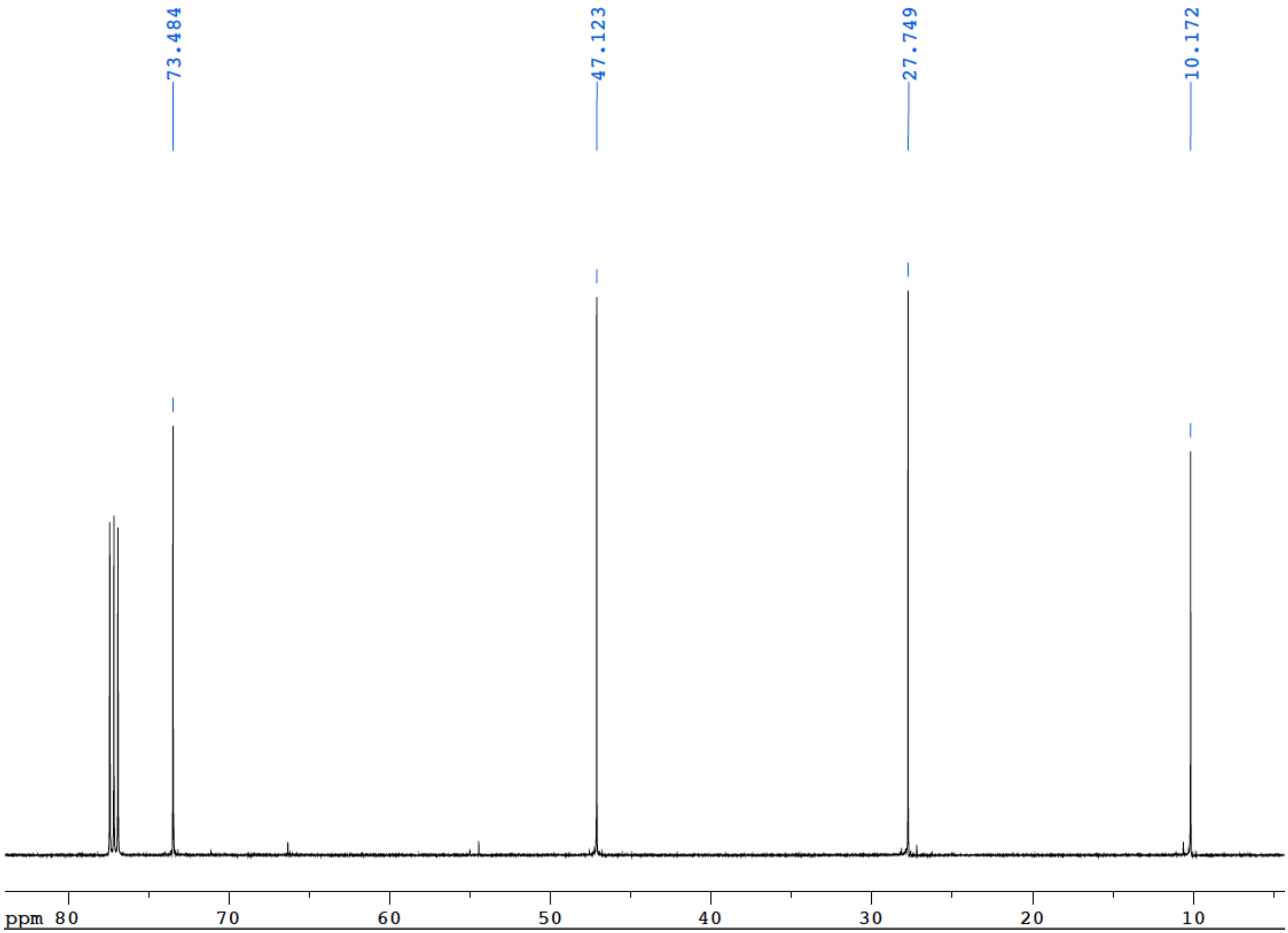

Figure S5. ^{1}H NMR Spectrum of (*R*)-**7b** ($CDCl_3$, 500 MHz).

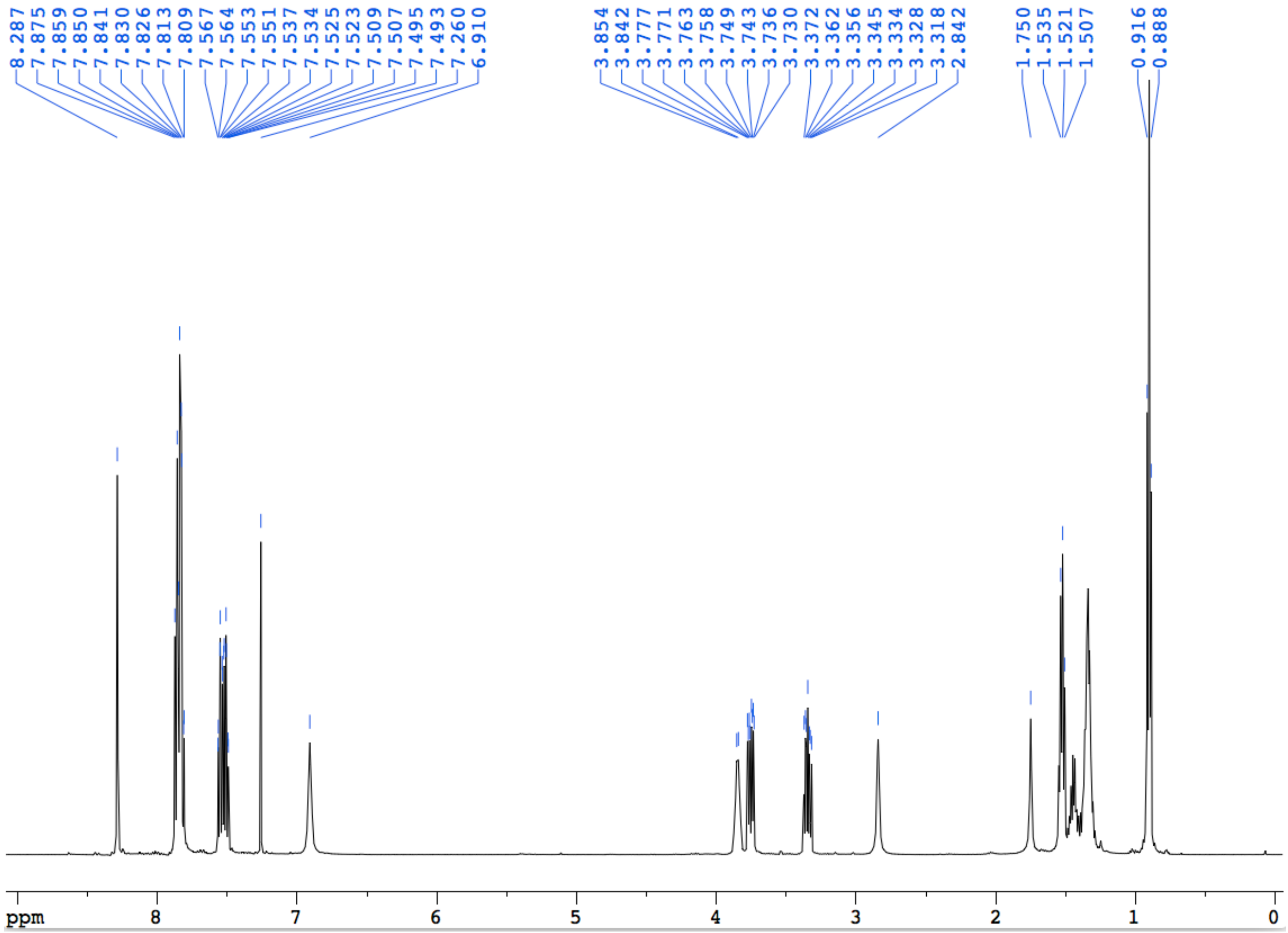

Figure S6. ^{13}C NMR Spectrum of (*R*)-**7b** ($CDCl_3$, 125 MHz).

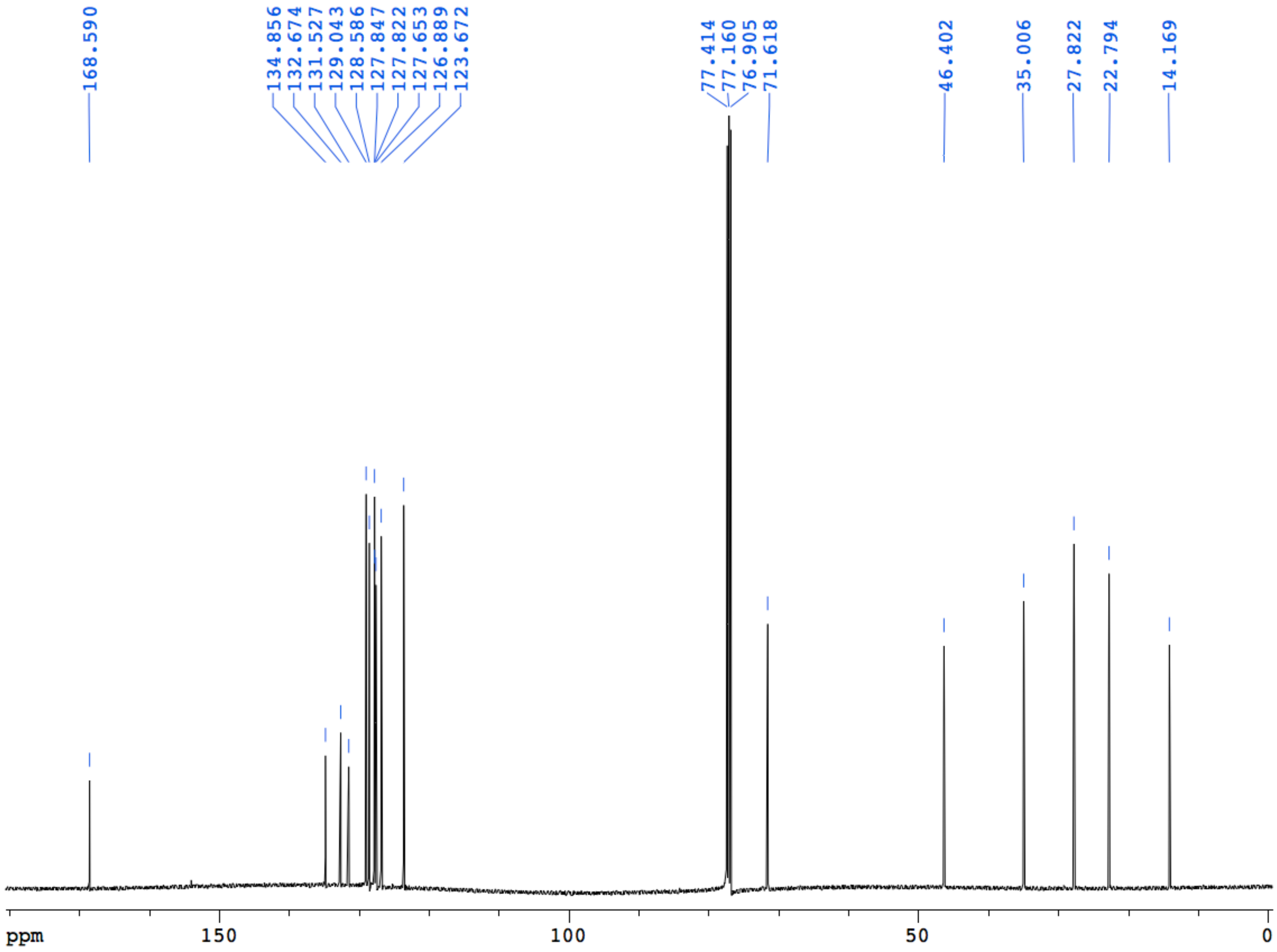

Figure S7. ^{1}H NMR Spectrum of **8** ($CDCl_3$, 500 MHz)

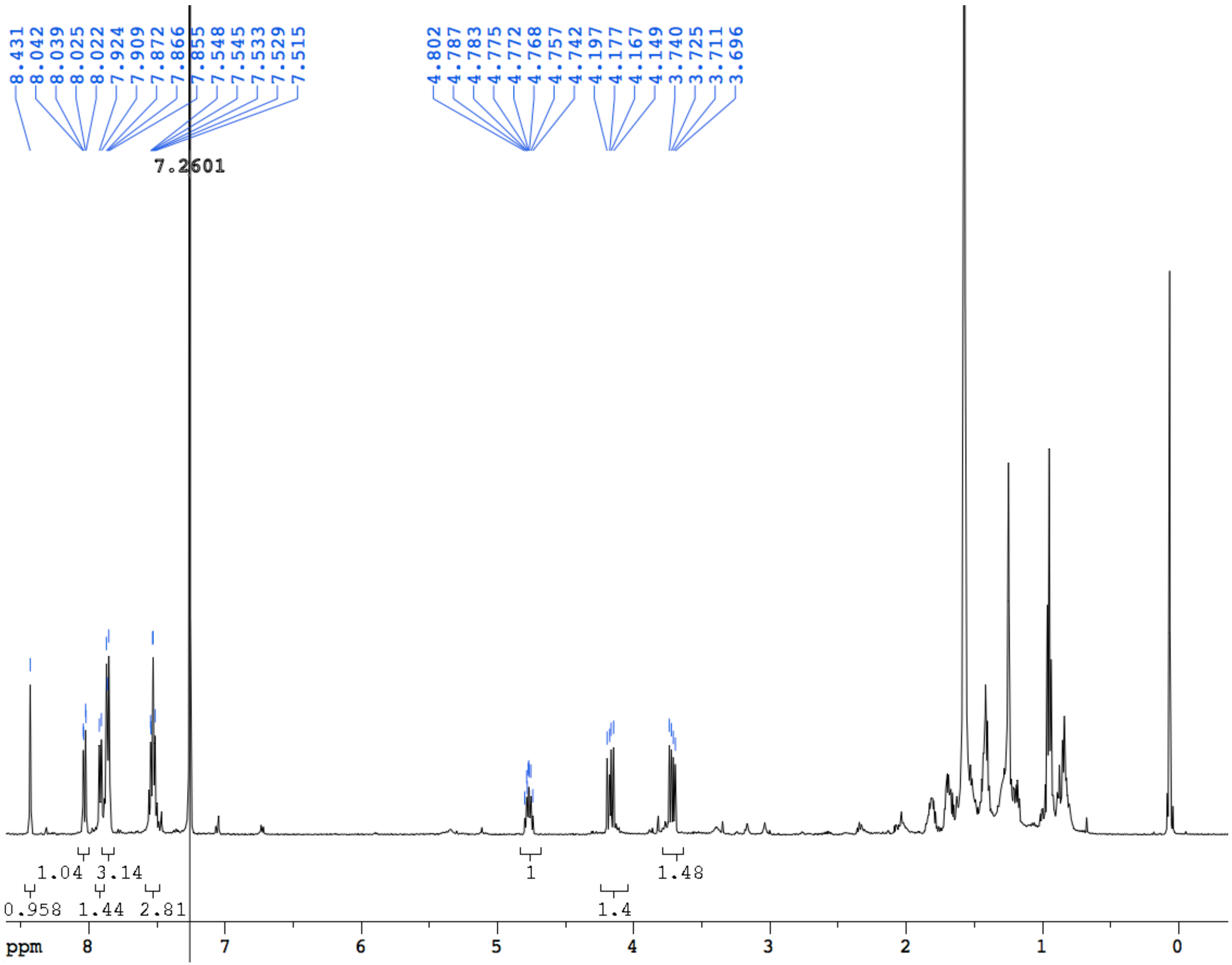

Figure S8. ^{1}H NMR Spectrum of (*R*)-**9b** ($CDCl_3$, 500 MHz) and expansions (a), (b), (c).

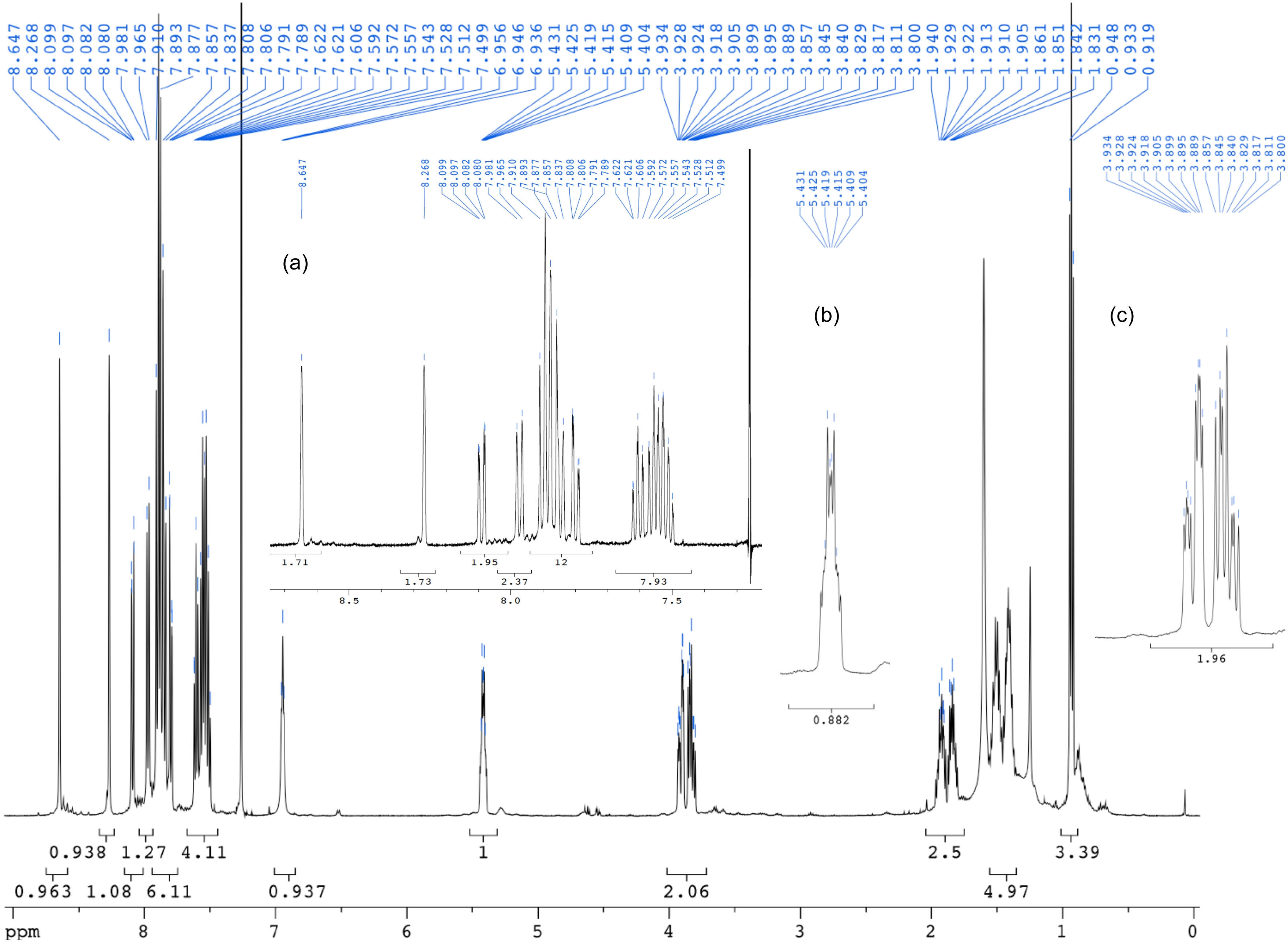

Figure S9. ^{13}C NMR Spectrum of (*R*)-**9b** ($CDCl_3$, 125 MHz).

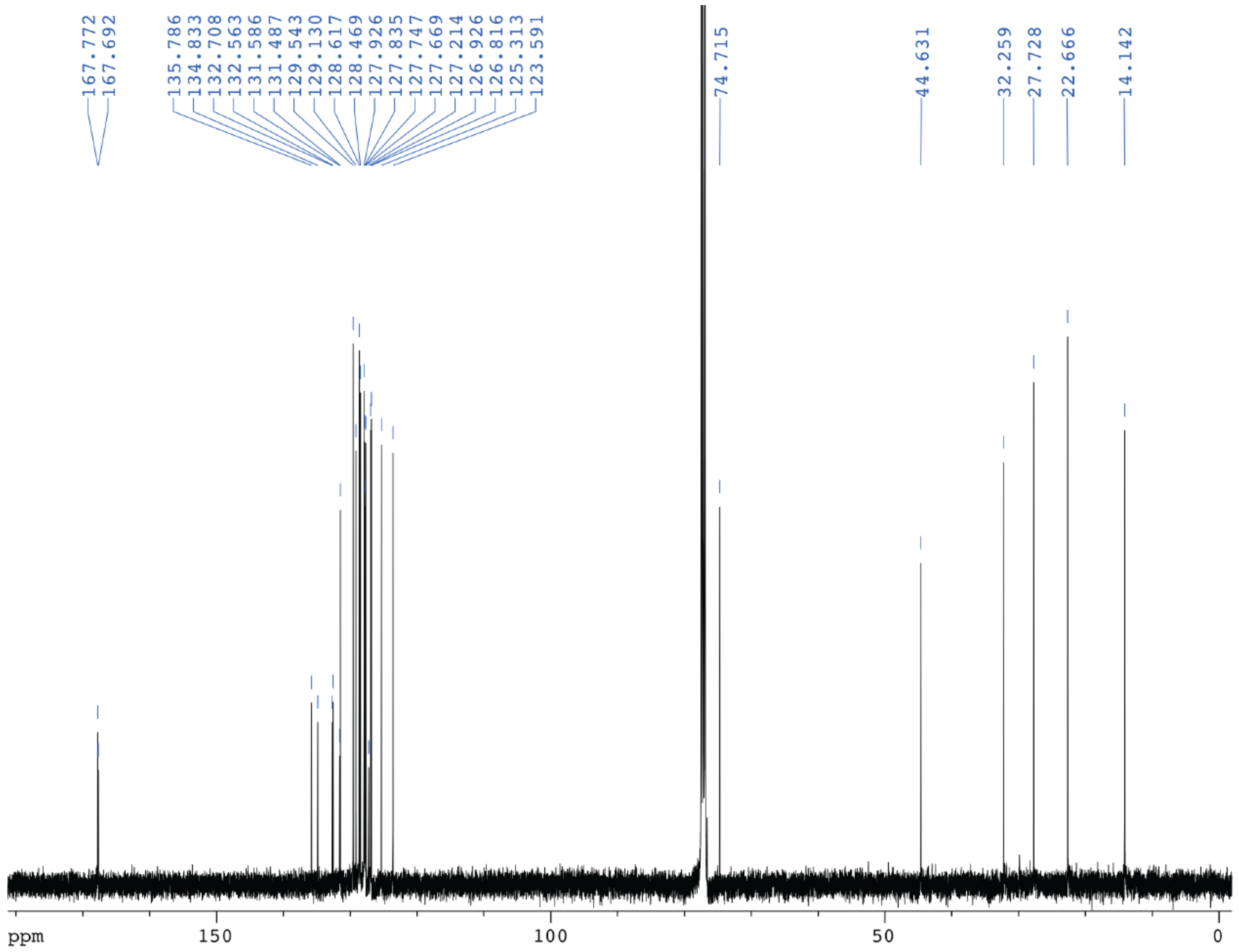

Figure S10. ^{1}H NMR Spectrum of (*S*)-**10a** ($CDCl_3$, 500 MHz)

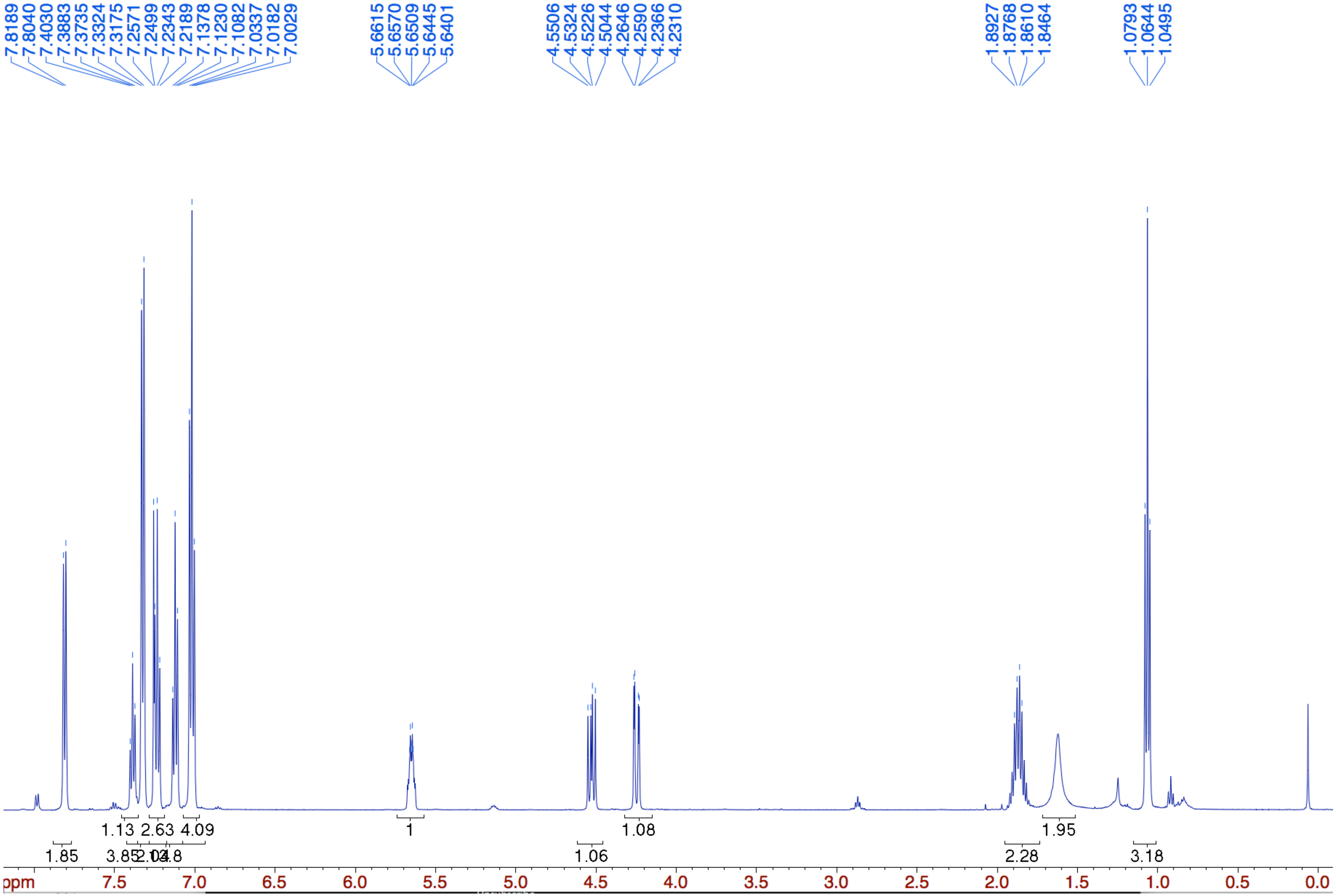

Figure S11. ^{13}C NMR Spectrum of (*S*)-**10a** ($CDCl_3$, 125 MHz).

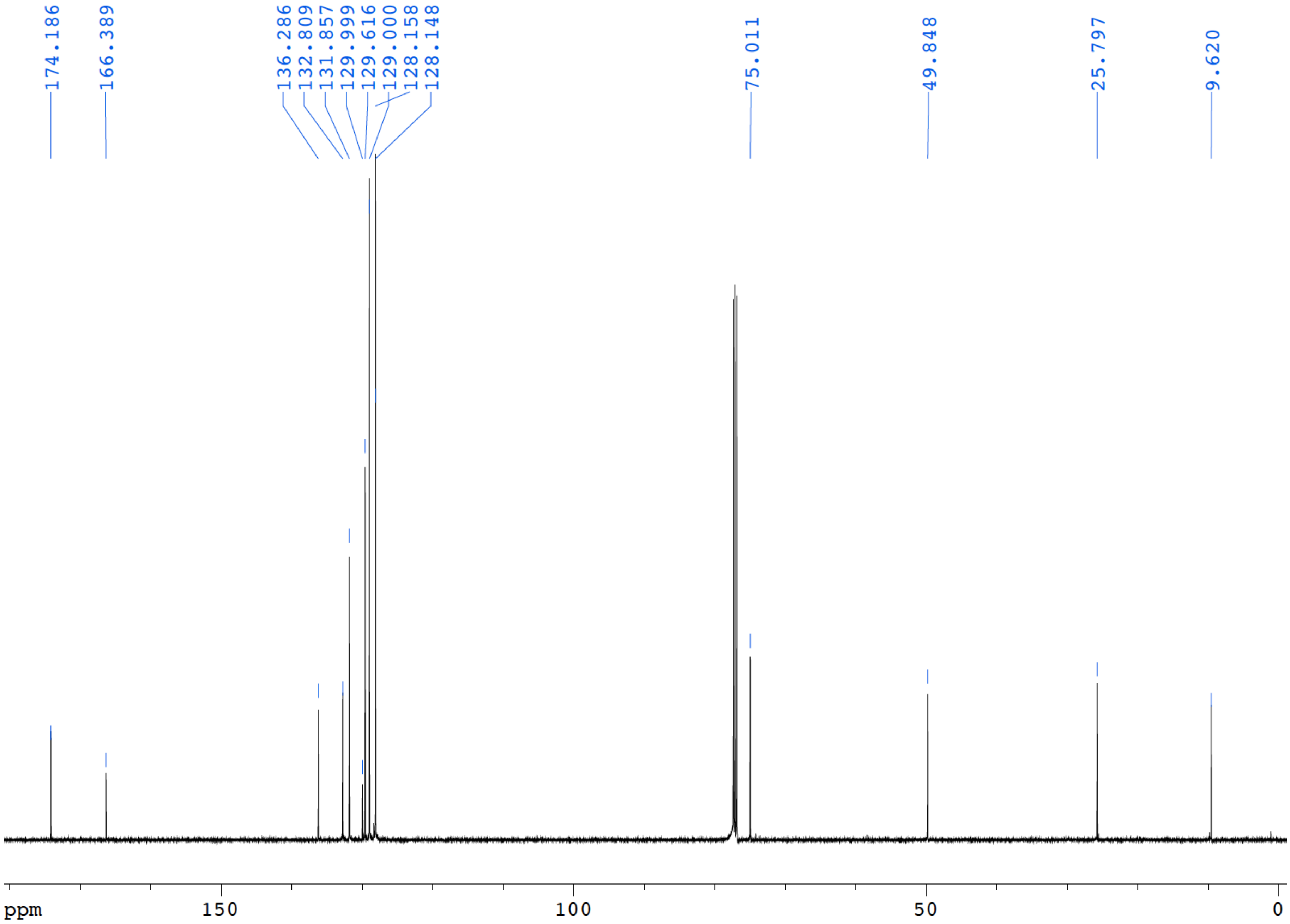

Figure S12. ^{1}H NMR Spectrum of (*R*)-**11b** ($CDCl_3$, 500 MHz).

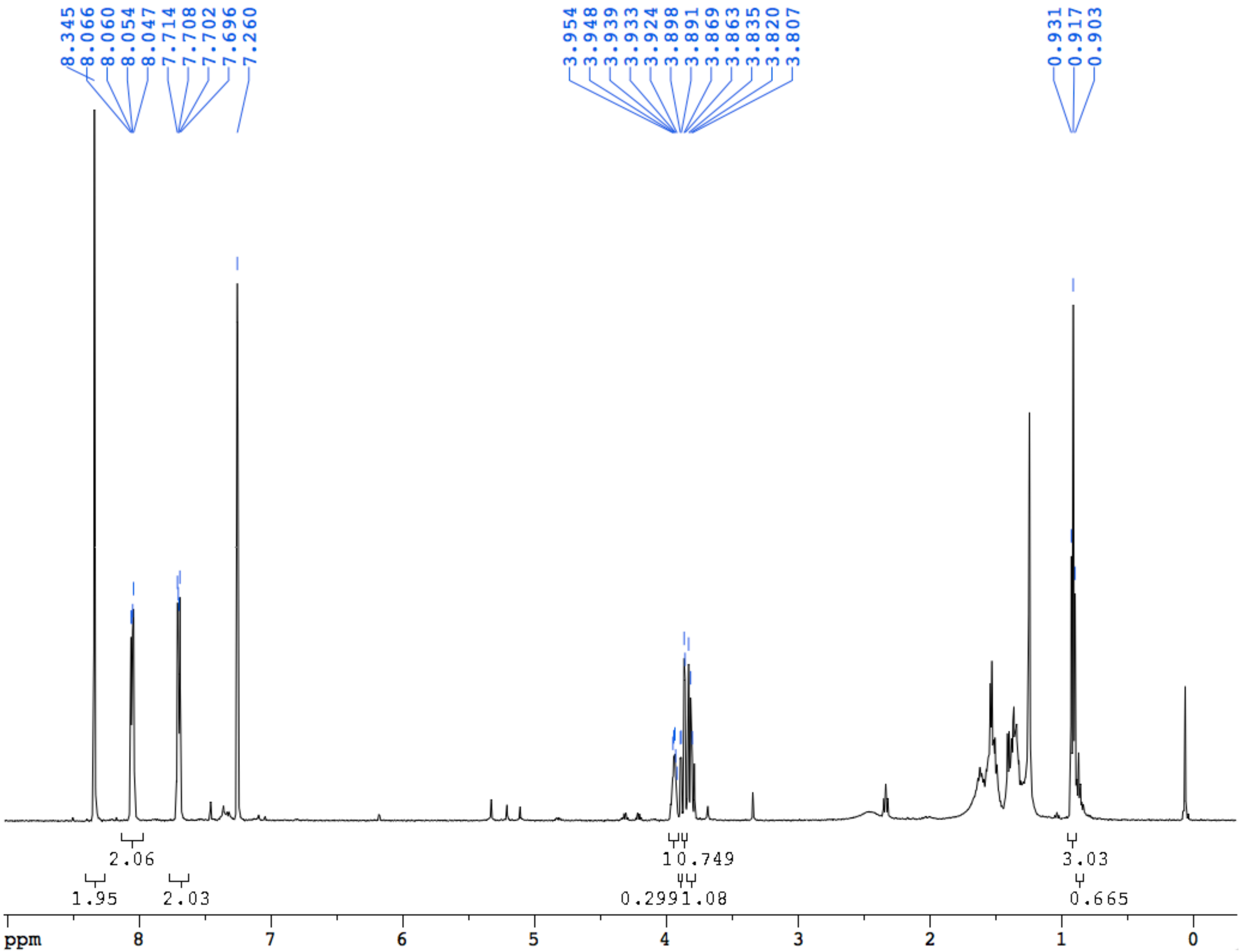

Figure S13. ^{13}C NMR Spectrum of (*R*)-**11b** ($CDCl_3$, 150 MHz).

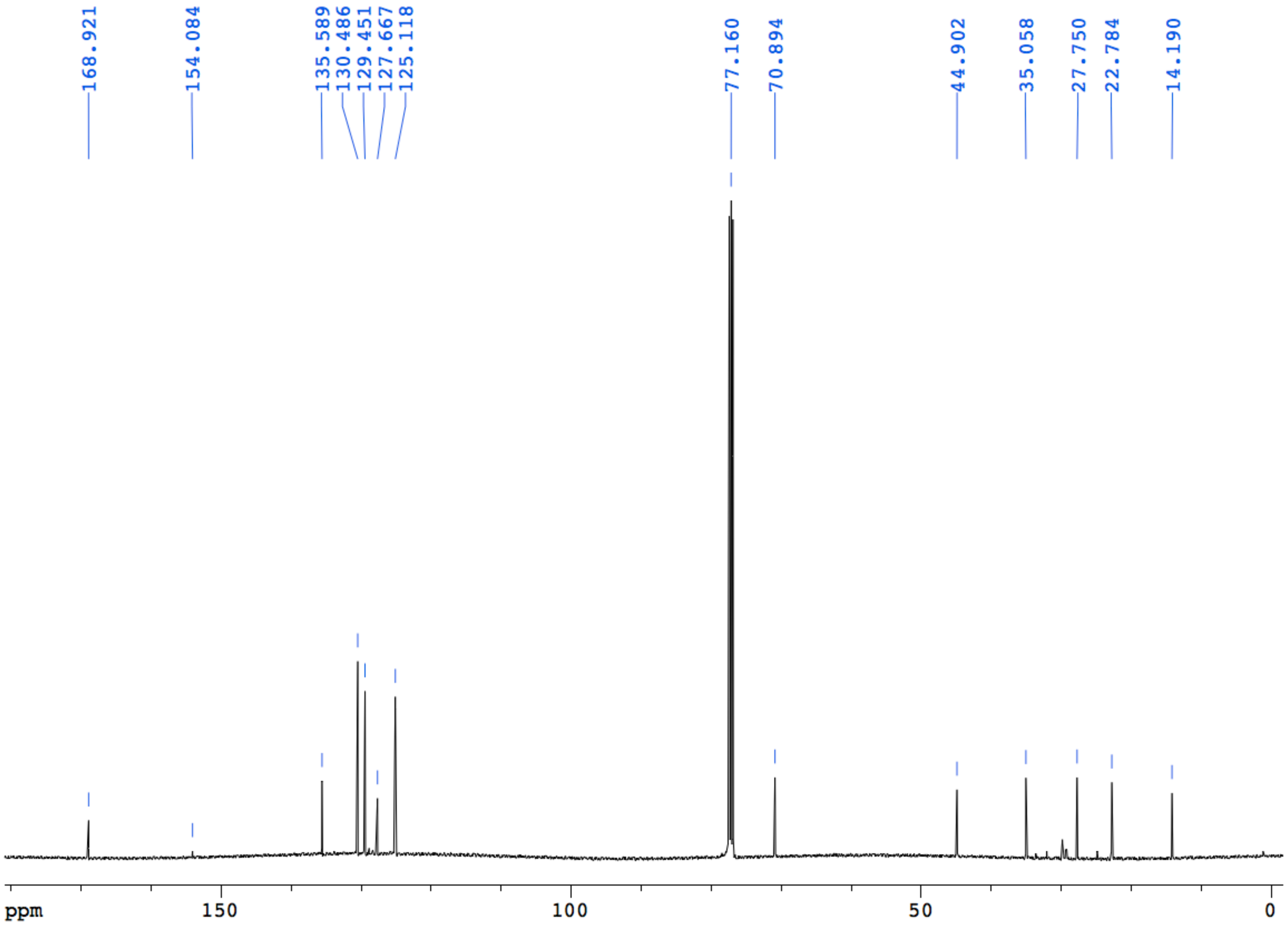

Figure S14. ^{1}H NMR Spectrum of (*R*)-**12b** ($CDCl_3$, 500 MHz).

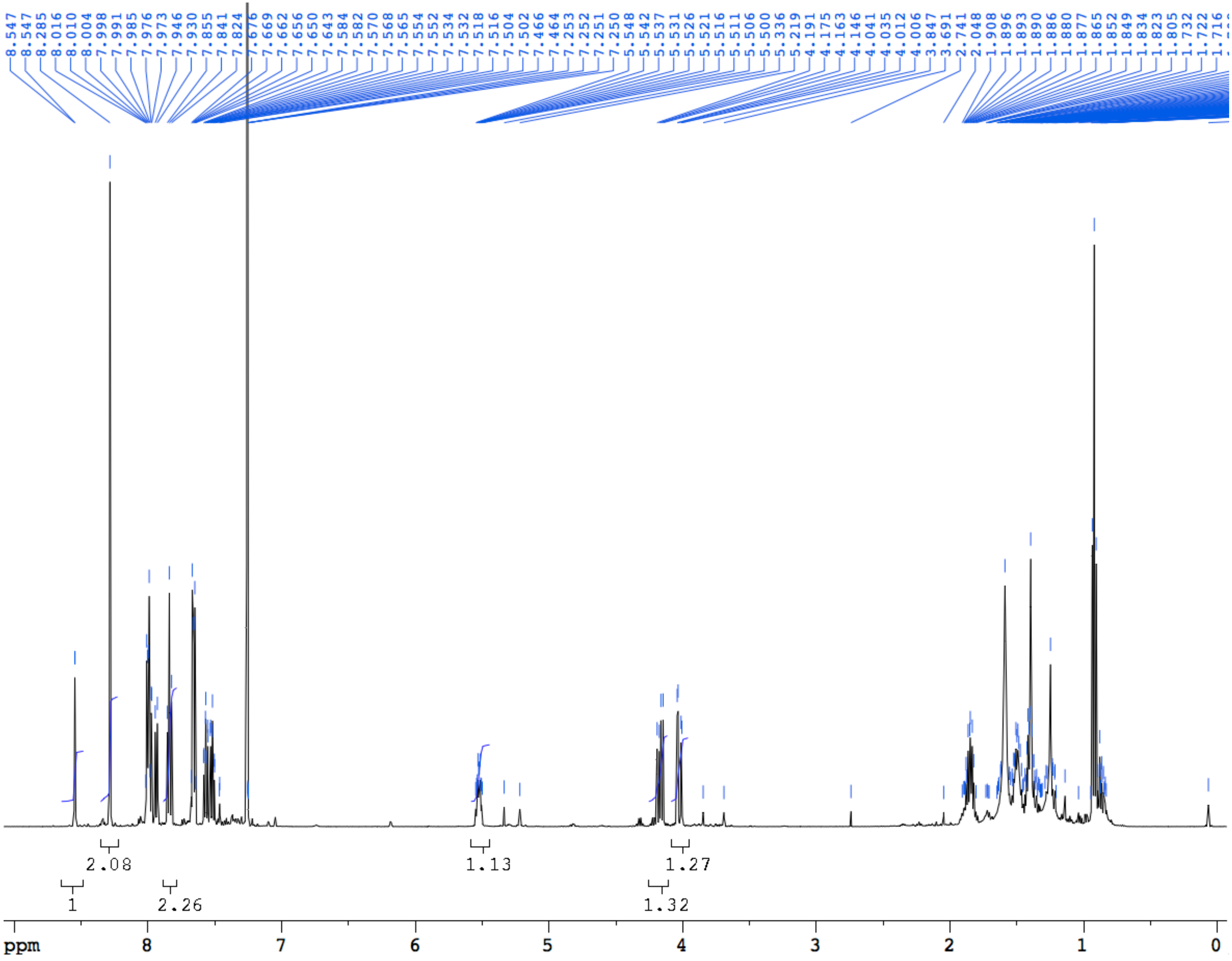

Figure S15. ^{1}H NMR Spectrum of (*R*)-**12b** ($CDCl_3$, 500 MHz) – Expansions (a) and (b)

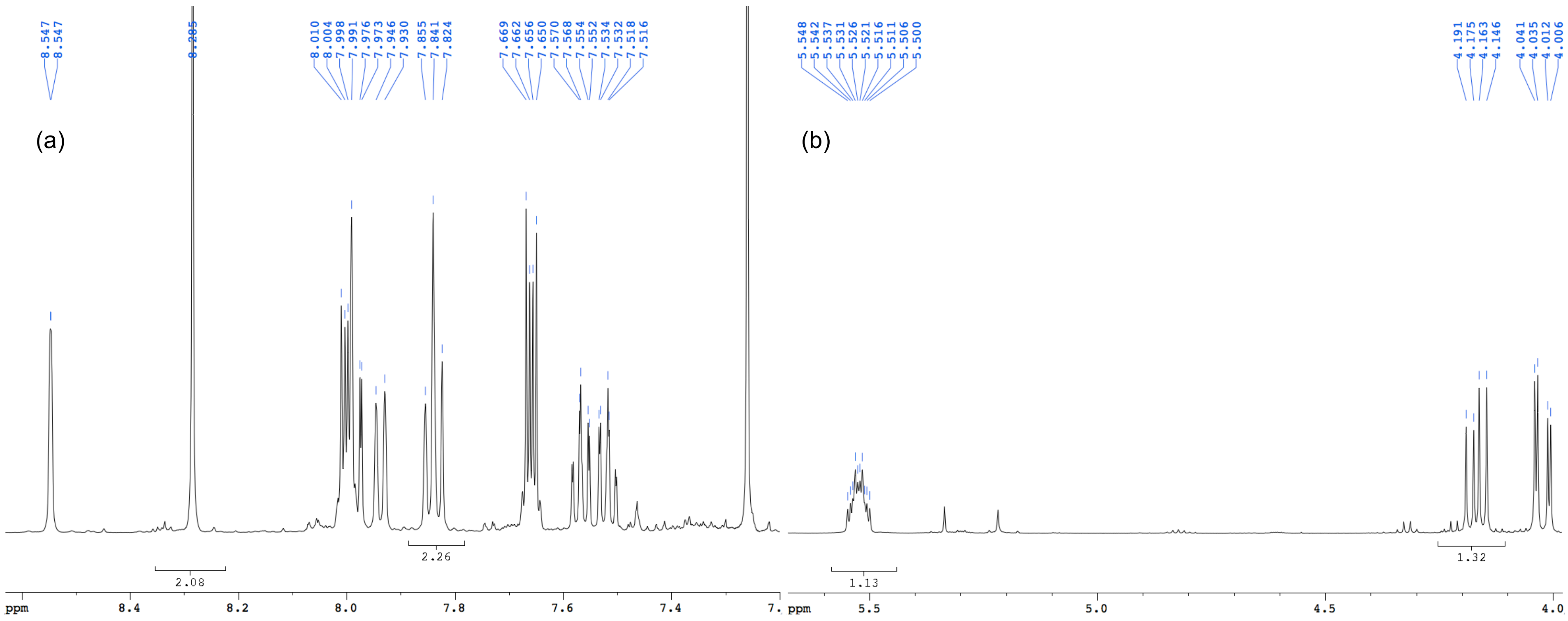

Figure S16. ^{1}H NMR Spectrum of (*S*)-**13a** ($CDCl_3$, 400 MHz)

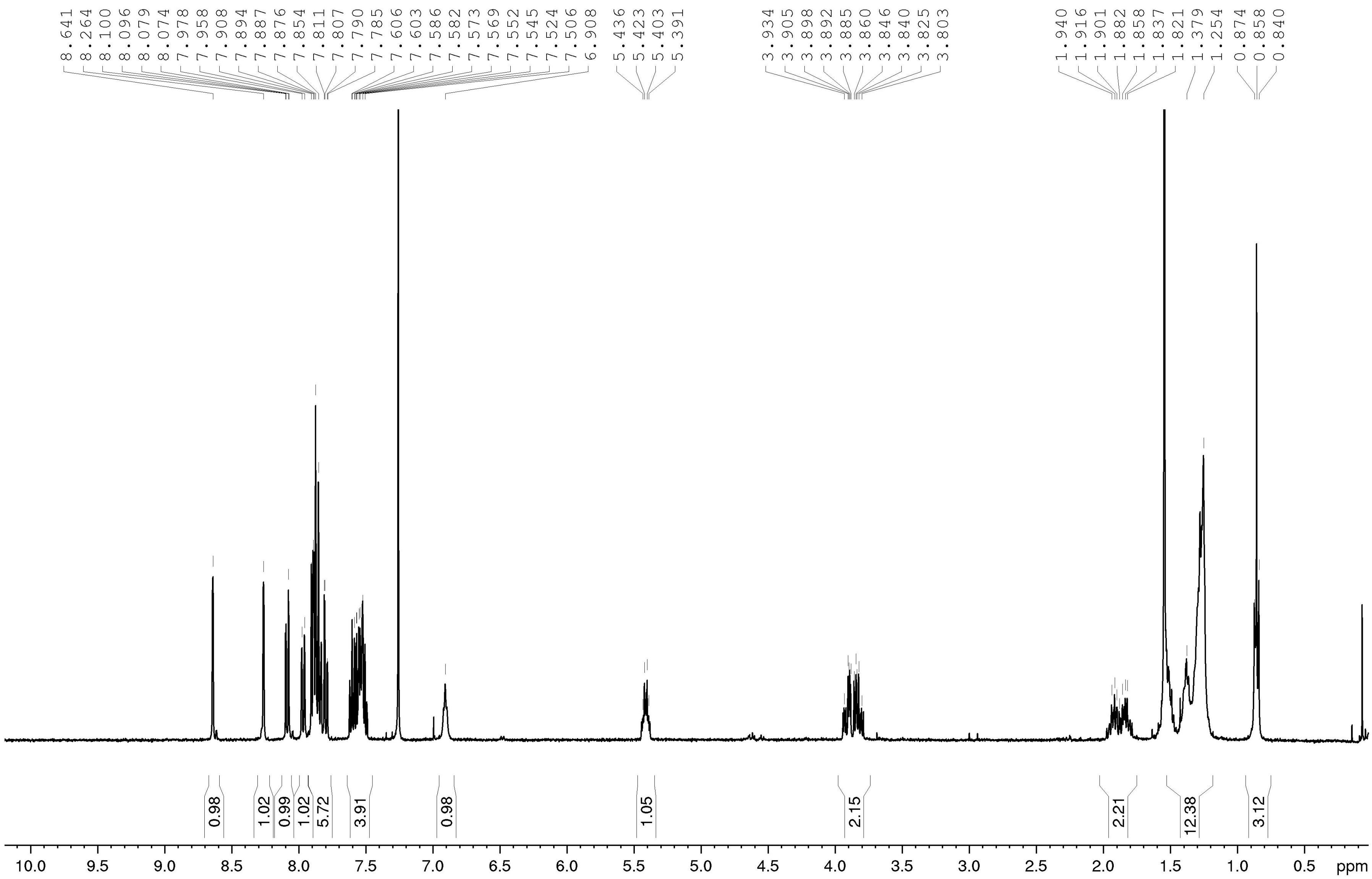

Figure S17. ESI HRMS of (a) (*R*)-**5b**, (b) (*S*)-**6a** and (c) (*R*)-**7b**.

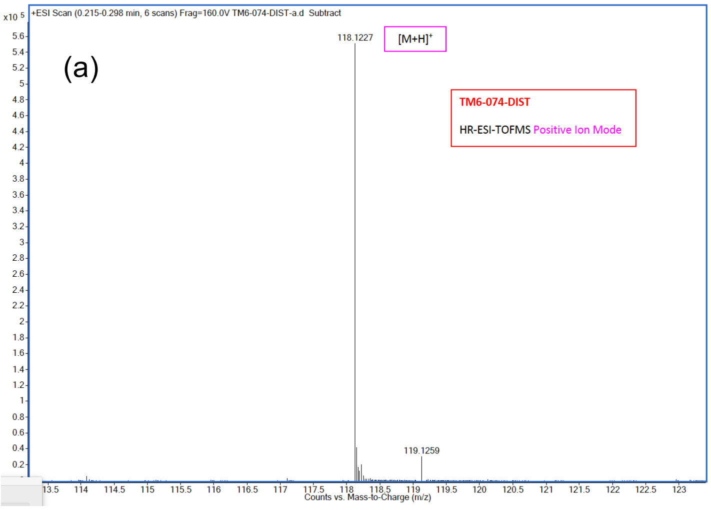

Search Results: Sample TM6-074-DIST

Mass Measured	Theo. Mass	Delta (ppm)	Composition
118.1227	118.1226	0.8	$[C_6H_{16}NO]^+$

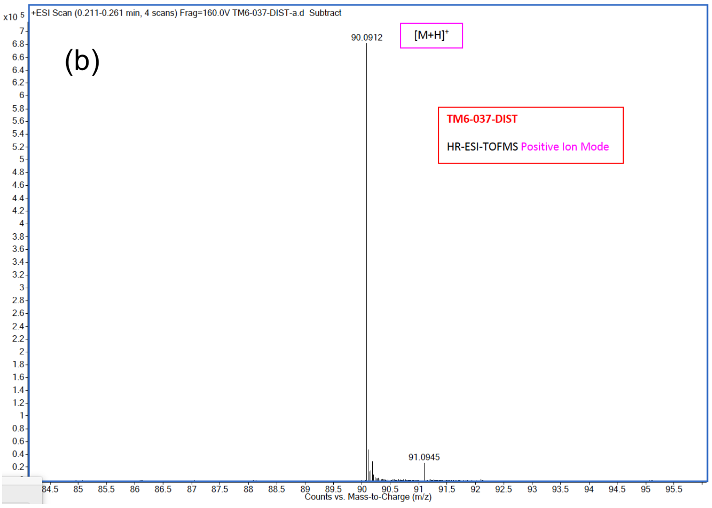

Search Results: Sample TM6-037-DIST

Mass Measured	Theo. Mass	Delta (ppm)	Composition
90.0912	90.0913	-1.1	$[C_4H_{12}NO]^+$

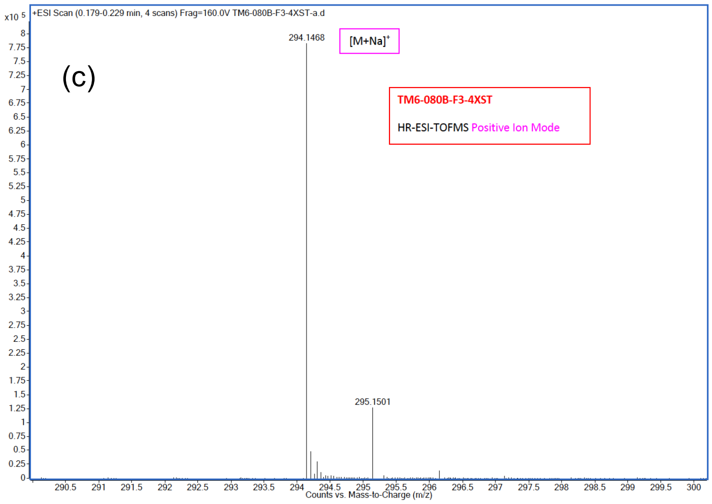

Search Results: Sample TM6-080B-F3-4XST

Mass Measured	Theo. Mass	Delta (ppm)	Composition
294.1468	294.1465	1.0	$[C_{17}H_{21}NO_2Na]^+$

Figure S18. ESI HRMS of (a) **8**, (b) (*R*)-**9b** and (c) (*S*)-**10a**.

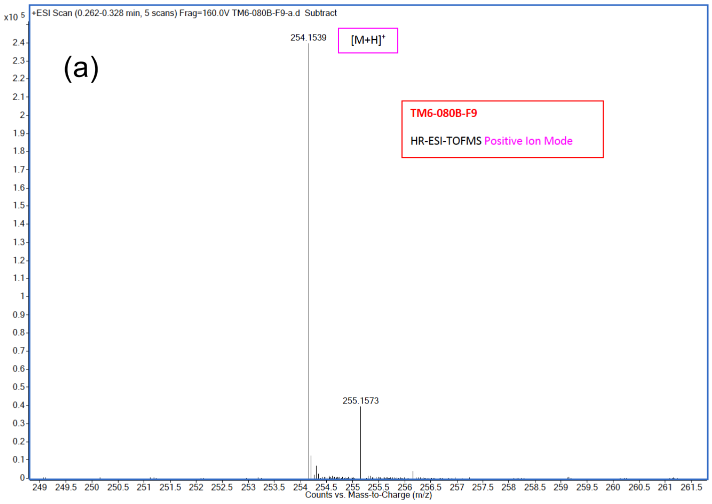

Search Results: Sample TM6-080B-F9

Mass Measured	Theo. Mass	Delta (ppm)	Composition
254.1539	254.1539	0.0	$[C_{17}H_{20}NO]^+$

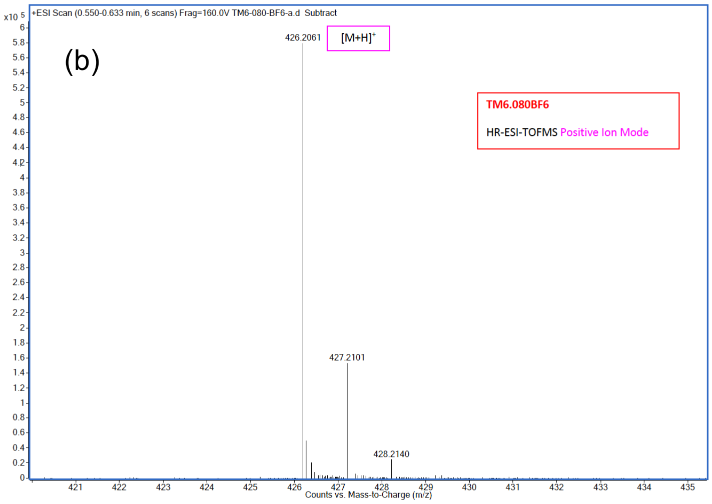

Search Results: Sample TM6.080BF6

Mass Measured	Theo. Mass	Delta (ppm)	Composition
426.2061	426.2064	-0.7	$[C_{28}H_{28}NO_3]^+$

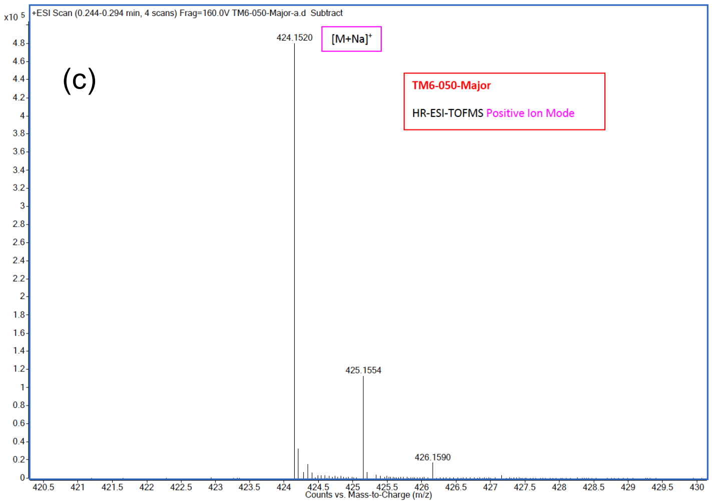

Search Results: Sample TM6-050-Major

Mass Measured	Theo. Mass	Delta (ppm)	Composition
424.1520	424.1519	0.2	$[C_{25}H_{23}NO_4Na]^+$

Figure S19. ESI HRMS of (a) (*R*)-**11b**, (b) (*R*)-**12b** and (c) (*S*)-**13a**.

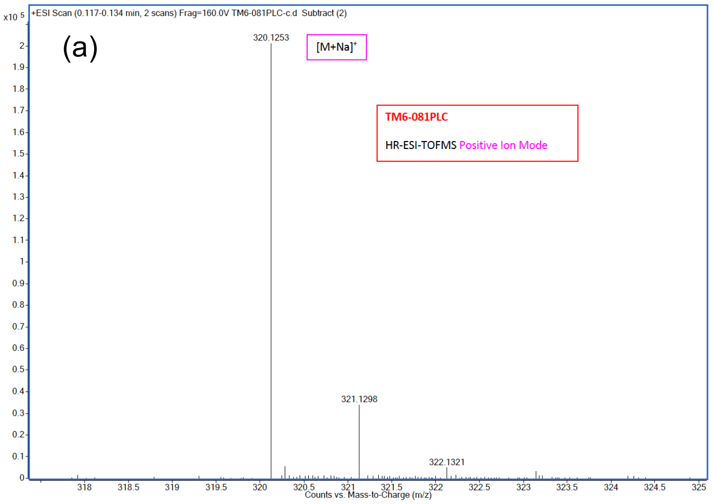

Search Results: Sample TM6-081PLC

Mass Measured	Theo. Mass	Delta (ppm)	Composition
320.1253	320.1257	-1.3	$[C_{18}H_{19}NO_3Na]^+$

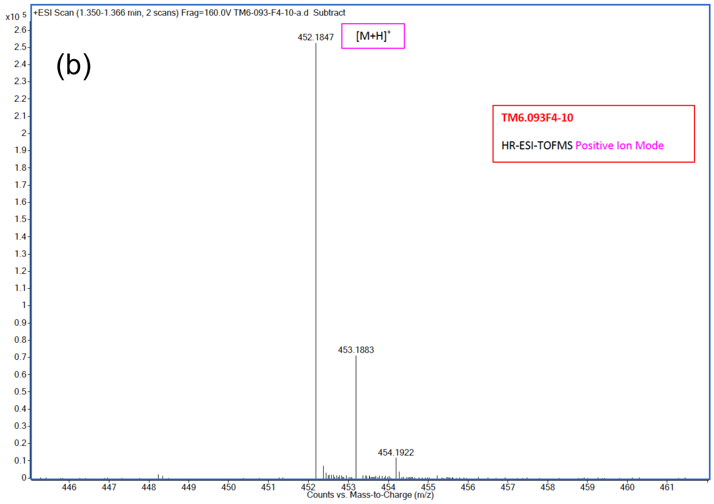

Search Results: Sample TM6.093F4-10

Mass Measured	Theo. Mass	Delta (ppm)	Composition
452.1847	452.1856	-2.0	$[C_{29}H_{26}NO_4]^+$

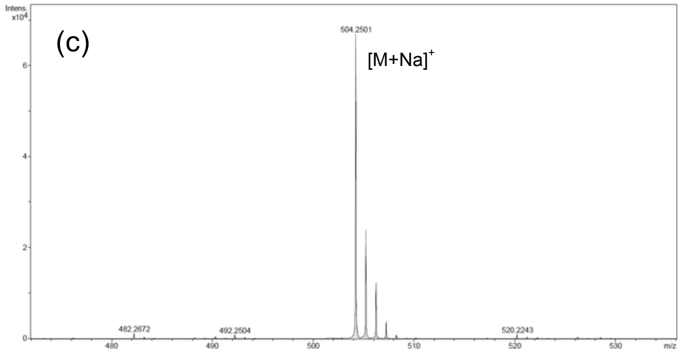

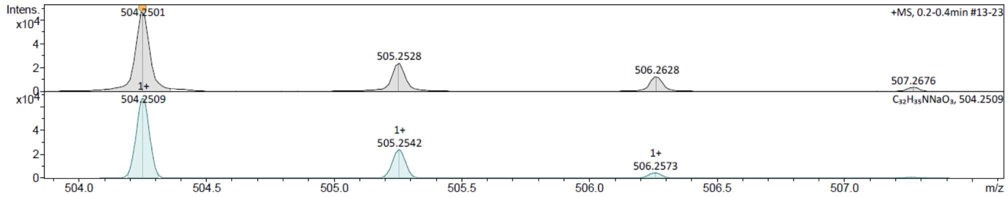

Meas. m/z	#	Ion Formula	m/z	err [ppm]	mSigma	# Sigma	Score	rdb	e⁻ Conf	N-Rule
504.2501	1	C32H32N4O2	504.2520	-3.7	62.5	1	100.00	19.0	odd	ok
	1	C32H32N4O2	504.2520	-3.7	62.5	1	100.00	19.0	odd	ok
	1	C32H35NNaO3	504.2509	-1.6	63.1	1	100.00	15.5	even	ok

Figure S20. ^{1}H NMR Spectrum of bis (*S*)-MPA derivative of (*S*)-**3a** ($CDCl_3$, 500 MHz).

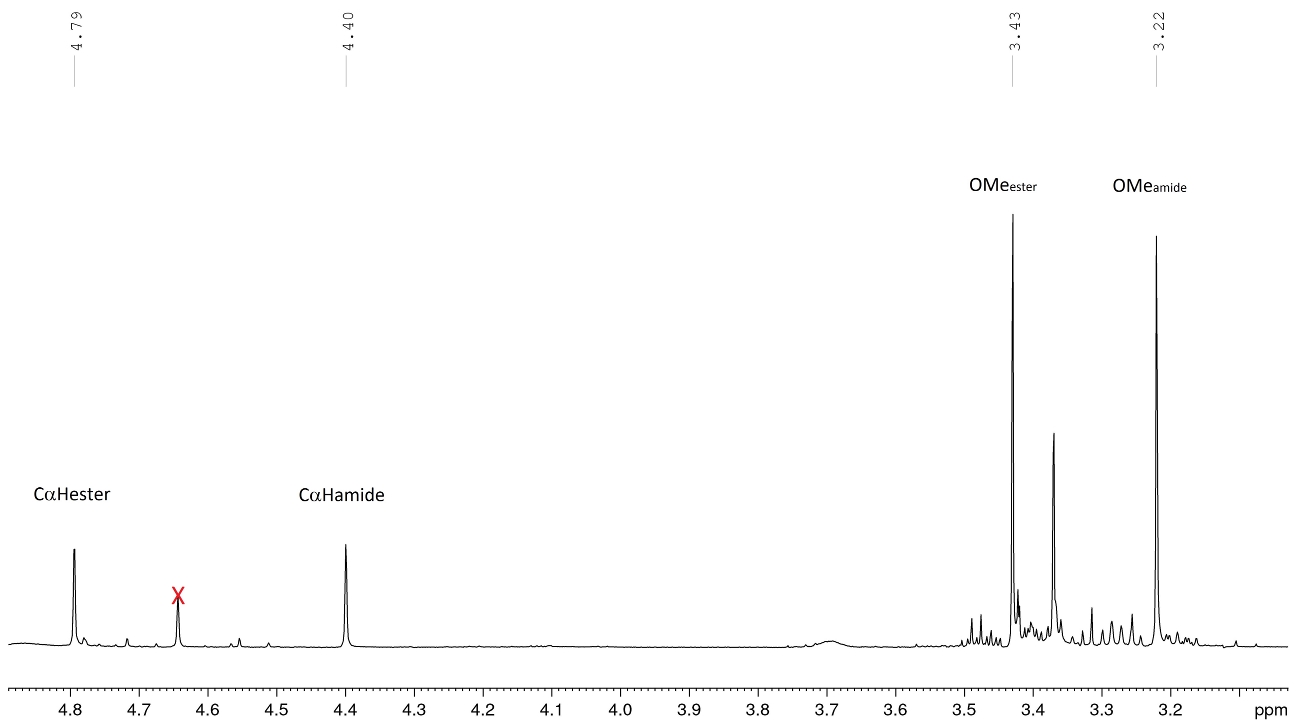

Figure S21. ^{1}H NMR Spectra of (a) *bis*-(*S*)-MPA **1c** and (b) *bis*-(*R*)-MPA **1d** highlighting CαH resonances and integrals ($CDCl_3$, 500 MHz).

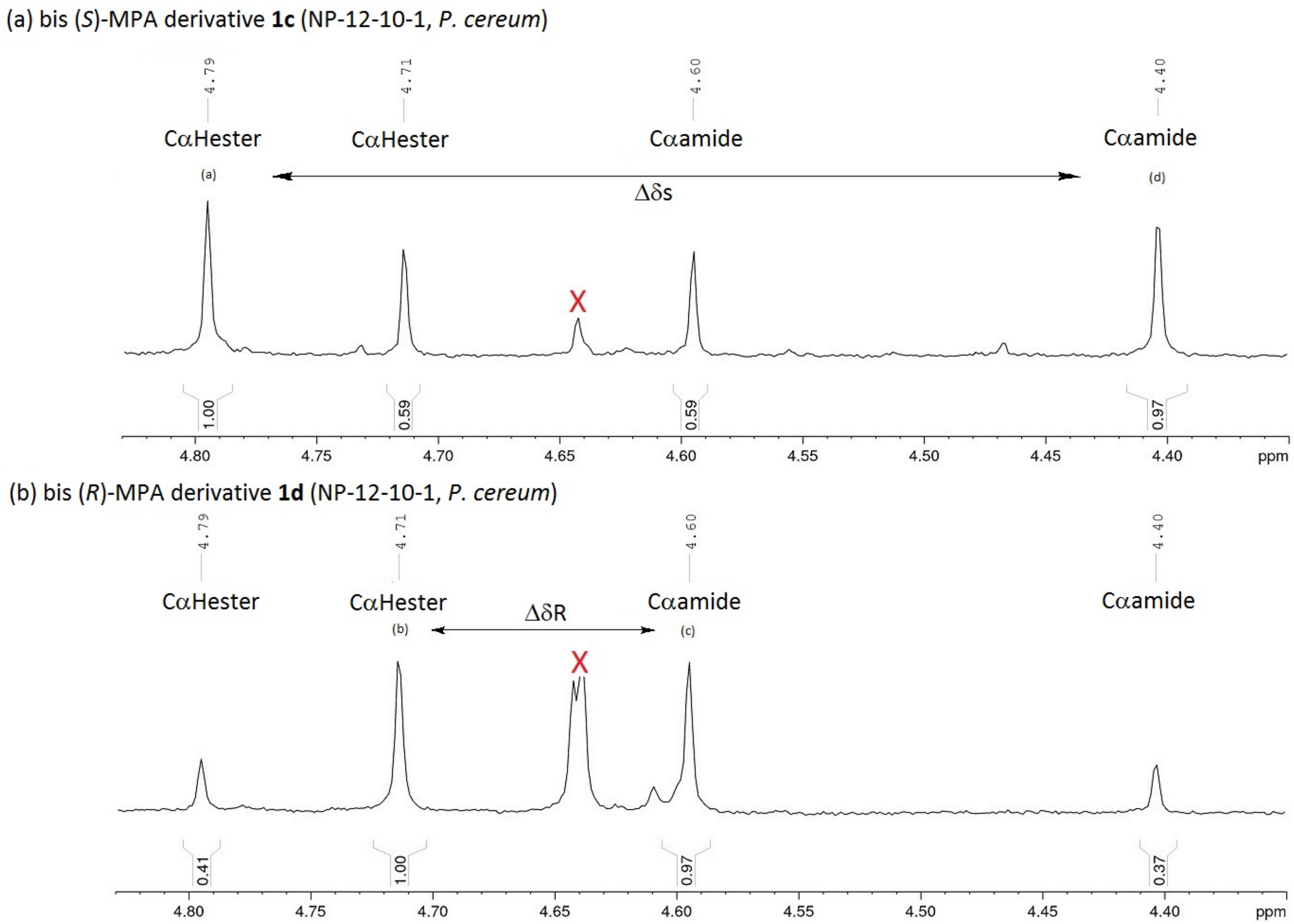

Supplement: Supplementary file 1 [file molecules-24-00090-s001.pdf]
